# Interfacial molecular interactions of cellobiohydrolase Cel7A and its variants on cellulose

**DOI:** 10.1186/s13068-020-1649-7

**Published:** 2020-01-18

**Authors:** Akshata R. Mudinoor, Peter M. Goodwin, Raghavendra U. Rao, Nardrapee Karuna, Alex Hitomi, Jennifer Nill, Tina Jeoh

**Affiliations:** 1 Biological and Agricultural Engineering, University of California, Davis, One Shields Ave., Davis, CA 95616 USA; 2 Center for Integrated Nanotechnologies, Los Alamos National Laboratory, Los Alamos, NM 87545 USA; 3 Gracenote, Inc., 2000 Powell Street, Suite 1500, Emeryville, CA 94608 USA; 4 Biotechnology, Faculty of Engineering and Industrial Technology, Silpakorn University, Nakhon Pathom, 73000 Thailand; 5 Chemical Engineering, University of California, Davis, One Shields Ave., Davis, CA 95616 USA; 6 Molecular Biophysics and Integrated Bioimaging, Lawrence Berkeley National Lab, 1 Cyclotron Road, Berkeley, CA 94720 USA

**Keywords:** *Trichoderma reesei* Cel7A, Super-resolution, Single-molecule imaging, Catalytic domain, Binding lifetime, Dissociation rate, Heterogeneous interfacial enzyme kinetics

## Abstract

**Background:**

Molecular-scale mechanisms of the enzymatic breakdown of cellulosic biomass into fermentable sugars are still poorly understood, with a need for independent measurements of enzyme kinetic parameters. We measured binding times of cellobiohydrolase *Trichoderma reesei* Cel7A (Cel7A) on celluloses using wild-type Cel7A (WT_intact_), the catalytically deficient mutant Cel7A E212Q (E212Q_intact_) and their proteolytically isolated catalytic domains (CD) (WT_core_ and E212Q_core_, respectively). The binding time distributions were obtained from time-resolved, super-resolution images of fluorescently labeled enzymes on cellulose obtained with total internal reflection fluorescence microscopy.

**Results:**

Binding of WT_intact_ and E212Q_intact_ on the recalcitrant algal cellulose (AC) showed two bound populations: ~ 85% bound with shorter residence times of < 15 s while ~ 15% were effectively immobilized. The similarity between binding times of the WT and E212Q suggests that the single point mutation in the enzyme active site does not affect the thermodynamics of binding of this enzyme. The isolated catalytic domains, WT_core_ and E212Q_core_, exhibited three binding populations on AC: ~ 75% bound with short residence times of ~ 15 s (similar to the intact enzymes), ~ 20% bound for < 100 s and ~ 5% that were effectively immobilized.

**Conclusions:**

Cel7A binding to cellulose is driven by the interactions between the catalytic domain and cellulose. The cellulose-binding module (CBM) and linker increase the affinity of Cel7A to cellulose likely by facilitating recognition and complexation at the substrate interface. The increased affinity of Cel7A to cellulose by the CBM and linker comes at the cost of increasing the population of immobilized enzyme on cellulose. The residence time (or inversely the dissociation rates) of Cel7A on cellulose is not catalysis limited.
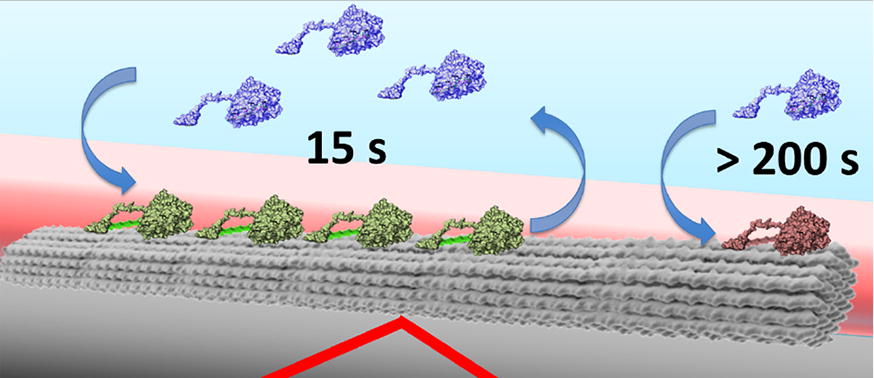

## Background

The population of the world is projected to exceed 9 billion by the year 2050, leading to 50% more demand for petroleum based liquid fuels that currently power the transportation sector [1]. Biofuels derived primarily from the most abundant biopolymer cellulose are a promising substitute for fossil fuels due to lowered greenhouse gas emissions, reduced climate change and health costs associated with their use [2]. However, the process of releasing soluble sugars from cellulose, a major component of the plant cell wall matrix is challenging. Cellulose, crystalline assemblies of β-1,4 linked glucose, is a very recalcitrant material and therein lies the challenge to the use of this substrate on a commercial scale [3].

Industrial cellulosic biofuel production processes employ fungal cellulase mixtures to breakdown cellulose into soluble sugars for further fermentation into fuels. In fungal cellulase mixtures, cellobiohydrolases (e.g., Cel7A of the well-characterized *Trichoderma reesei*) are the molecular workhorses that hydrolyze the recalcitrant cellulose in synergy with supporting endocellulase and oxidative activities [4, 5]. The processive hydrolysis of cellulose by *T. reesei* Cel7A (Cel7A) entails multiple sequential steps: adsorption of the enzyme to the cellulose surface, surface diffusion, complexation wherein the catalytic domain recognizes and engages the reducing end of a single molecule of cellulose within its active site tunnel, glycosidic bond hydrolysis to form cellobiose as the product, product expulsion from the active site and subsequent sliding along the molecule to release several consecutive cellobioses (processive hydrolysis), decomplexation and finally desorption from the cellulose surface [6]. Mechanistic kinetic models of cellulose hydrolysis suggest that the complexation and decomplexation steps are rate-limiting elementary cellulase–cellulose interactions and that cellulose hydrolysis rates are largely insensitive to the catalytic rate constant of the complexed enzymes [6].

The multi-modular structure of Cel7A, a 45–56 kDa catalytic domain (CD) and a ~ 4 kDa carbohydrate-binding module (CBM) connected by a ~ 10–15 kDa glycosylated linker, gives rise to multiple binding configurations of this enzyme on cellulose [7, 8] as all three domains have been shown to have affinity to cellulose [9–11]. Cel7A that is actively hydrolyzing cellulose must be complexed to cellulose by its CD; however, complexed Cel7A can stall and thereby become inactive [12, 13]. The different populations of bound Cel7A are challenging to distinguish in biochemical determinations of interaction rate parameters that are typically obtained from fitting hydrolysis or binding time courses, thereby resulting in broad ranges of values [14]. In one example, a dissociation rate constant of Cel7A (‘*k*_off_’) from crystalline and amorphous cellulose was estimated from hydrolysis curves to be 0.01–0.02 s^−1^ [15]. While another study measuring rates of insoluble reducing end formation reported Cel7A *k*_off_ = 0.0032 s^−1^ on bacterial microcrystalline cellulose and 0.007 s^−1^ on amorphous cellulose [16].

Single-molecule imaging is a means to directly measure desorption rates of cellobiohydrolases from cellulose [17–19]. Total internal reflection fluorescence microscopy (TIRFM) enables visualization of individual fluorescently labeled cellulases that approach within ~ 100 nm of the evanescent wave excited imaging surface. When isolated cellulose fibrils are deposited on the imaging surface, individual cellulases that bind to the fibril surfaces can be visualized [17]. A typical single-molecule imaging experiment records ‘movies’ consisting of multiple consecutive images over time. Analysis of the residence times of the cellulases observed to appear (bind) and disappear (unbind) from view (the cellulose surface) in the movie provides a measure of average binding lifetimes of the cellulases on cellulose (or ‘desorption rates’ from reciprocals of the binding times). This method has been used to determine various binding modes and desorption rates of Cel7A [17, 19] and Cel6A [18]. Despite similar enzymes, substrates and experimental setups, published studies of Cel7A report desorption rates that differ by 1–2 orders of magnitude.

In this study, we used super-resolution single-molecule imaging to measure the binding lifetimes of wild-type Cel7A purified from a commercial *T. reesei* enzyme mixture (WT_intact_) and the catalytically deficient mutant (E212Q_intact_) expressed in *T. reesei* on crystalline cellulose fibrils. A point mutation of the nucleophile Glu 212 to Gln 212 reduces the catalytic efficiency of Cel7A enzyme 2000-fold [20, 21] and provides a structurally intact Cel7A mutant to examine how catalysis impacts enzyme-binding lifetimes. Proteolytically isolated catalytic domains (WT_core_ and E212Q_core_) were also used to investigate binding specificity and lifetimes in the absence of the linker and binding modules. Unique to this study was the development of a robust and automated image analysis method to obtain binding lifetimes of all enzymes observed in the movies [22].

## Results

### Cellulose fibrils on the imaging surfaces

**Fig. 1:**
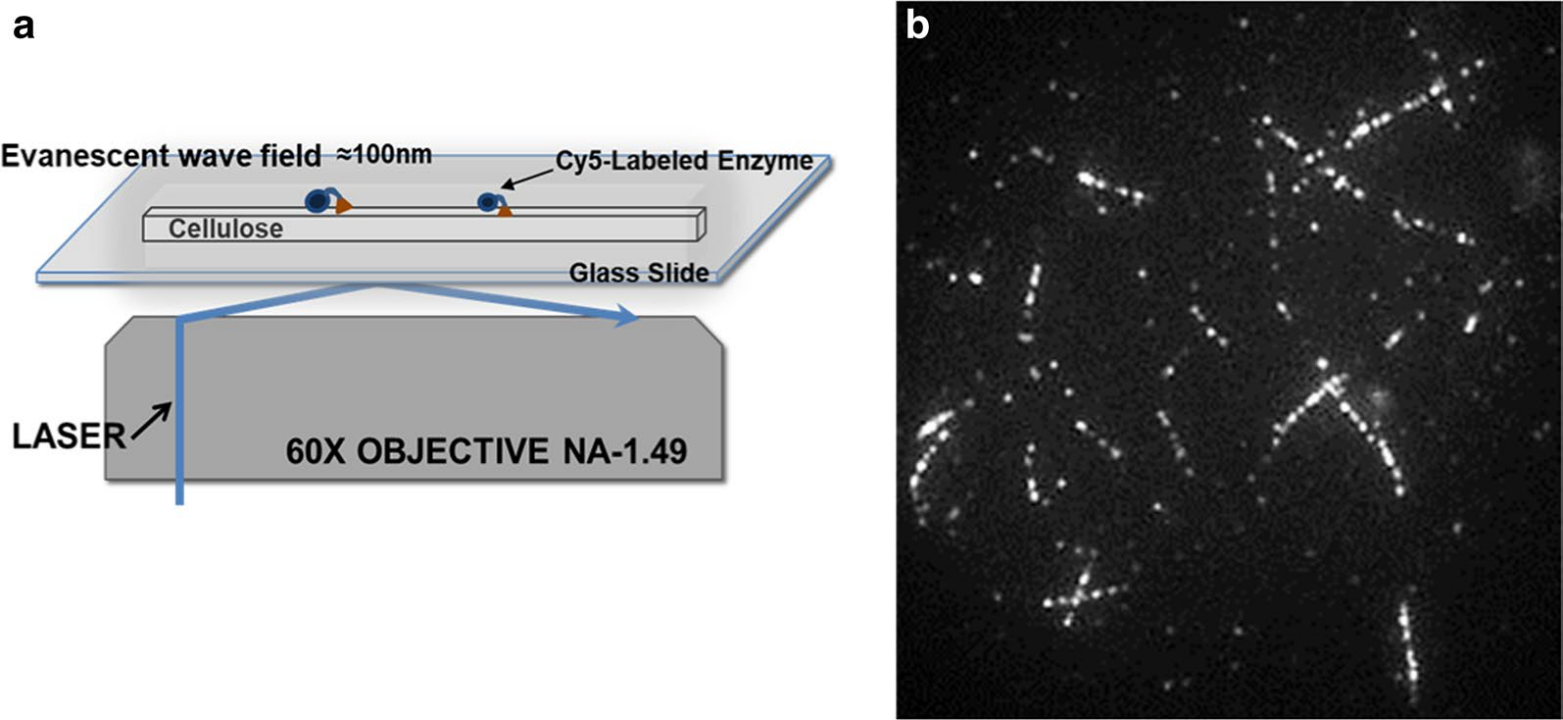
**a** A schematic illustration (not drawn to scale) of the through-objective TIRFM setup used to image Cy5-labeled enzymes bound to a cellulose fibril under evanescent wave excitation extending ~ 100 nm above the glass/water interface. **b** Cellulose fibrils with surface-bound Cy5-labeled cellulases were easily identifiable in the Cy5 fluorescence channel (56 × 56 μm^2^ field of view). Spots correspond to individual Cel7A enzymes. A single frame (1 s) of a 2500-frame data set is shown in **b**

The cellulose used in this study was a never-dried, highly crystalline algal cellulose (AC) isolated from cell walls of *C. aegagropila* and “polished” with a concentrated acid treatment. The polishing step reduced the productive Cel7A binding capacity (i.e., the number of Cel7A complexation sites per mass of cellulose) to 0.83 ± 0.13 µmol/g, which is considerably lower than either non-polished AC or other commercially available celluloses [13, 23]. Single-molecule binding experiments using non-polished AC and phosphoric acid swollen cellulose (PASC) suffered from heavily congested fibril surfaces where it was difficult to track individual molecules even when diminishingly low enzyme concentrations were used [24] (Additional file 1: S3 and S5). Acid polished AC (from here on referred to only as ‘AC’) significantly alleviated congestion of enzymes on the fibril surfaces (e.g., Fig. 1b).

**Fig. 2:**
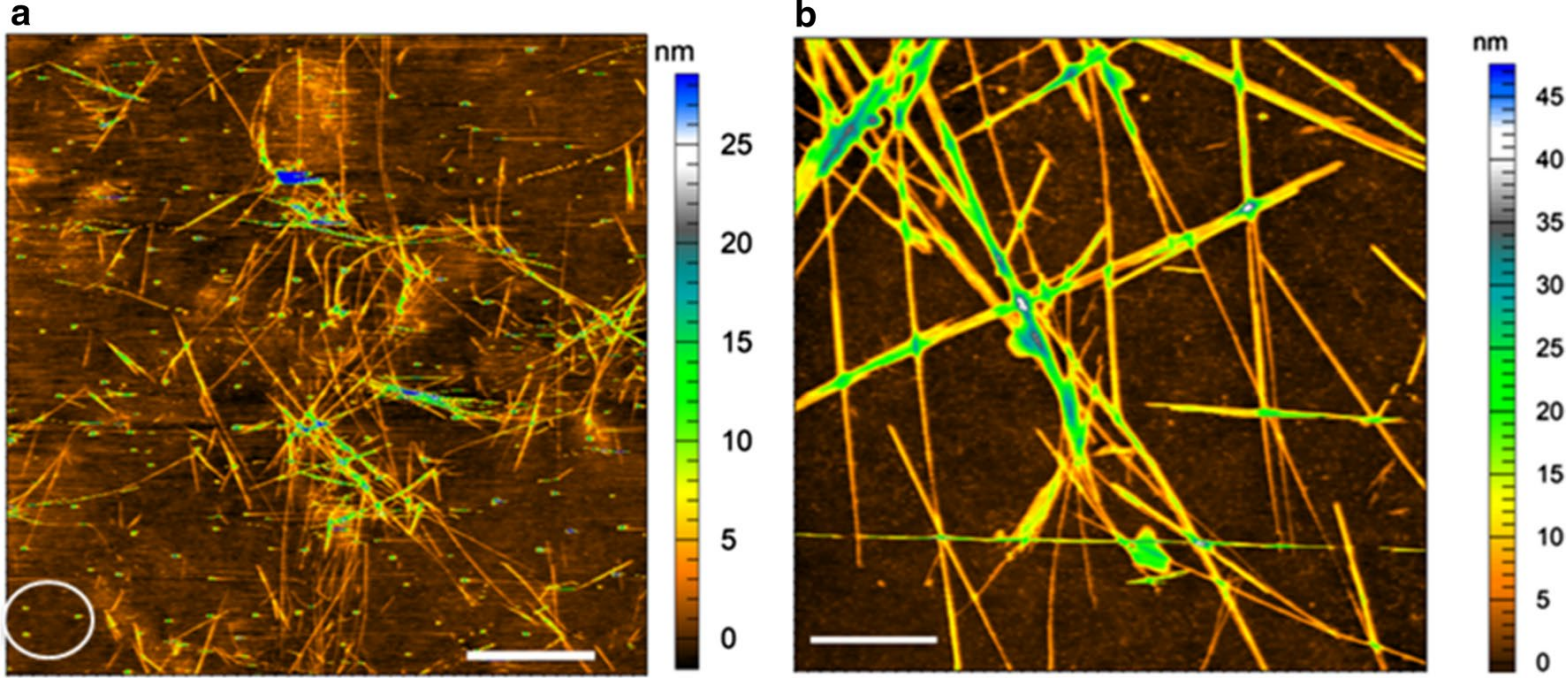
Atomic force microscopy (AFM) images of algal cellulose gravity-deposited on hydrophobically silanized glass imaging surfaces. **a** 50 × 50 µm^2^ field of view (scale bar = 10 µm), and **b** 5 × 5 µm^2^ field of view (scale bar = 1 µm). White circle in **a** highlights particles seen on the surface. Individual fibril heights ranged from ~ 3 to 6 nm

Individual and aggregated fibrils settled in random orientations on the surface of hydrophobically silanized glass (Fig. 2). Individual fibrils were tens of microns long and as thin as ~ 3–6 nm. Larger bundles, > 10 nm in height, were also common (e.g., Fig. 2b). Figure 2a exemplifies typical coverage of fibrils in a 50 × 50 µm^2^ area, a size comparable to the field of view obtained from TIRFM imaging in our setup. The white circle in (Fig. 2a) highlights particles (~ 30–40 nm high) commonly observed on the surfaces. While the composition of the particles is unknown, high phase contrasts from these particles in the AFM phase images suggest that these particles are more viscoelastic (i.e., ‘softer’) than the cellulose fibrils. One possibility is that these are nano-air bubbles trapped at the hydrophobized glass surface.

### Specificity of Cel7A to cellulose is determined by the carbohydrate-binding module (CBM)

**Fig. 3:**
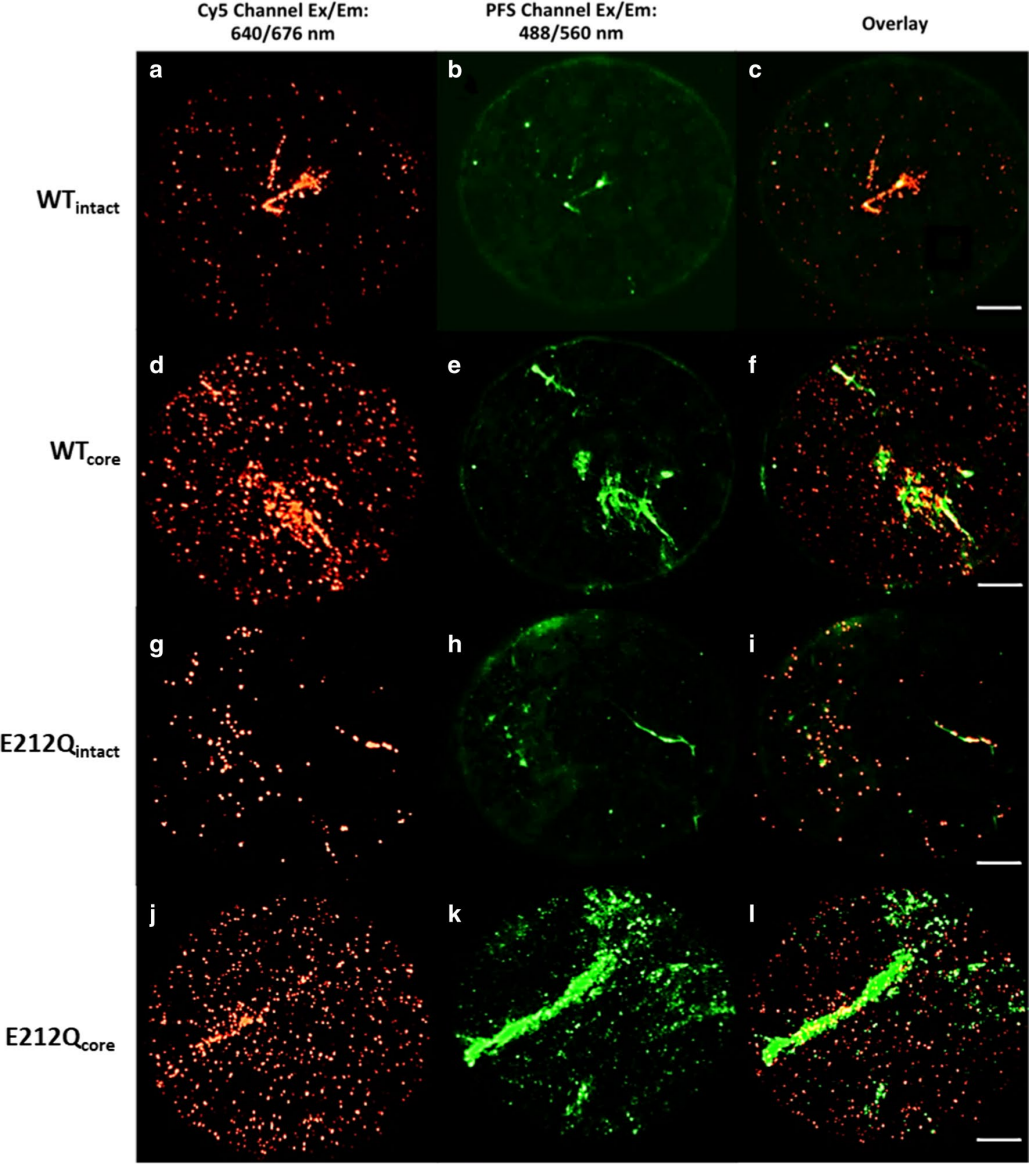
Binding to algal cellulose by WT_intact_ (**a**–**c**), WT_core_ (**d**–**f**) and E212Q_intact_ (**g**–**i**) and E212Q_core_ (**j**–**l**). False color images of Cy5-labeled cellulases binding to PFS-stained cellulose fibrils. Images in the left column (**a**, **d**, **g**, **j**) are of Cy5 emission excited at 637 nm, images in the middle column (**b**, **e**, **h**, **k**) are of PFS emission excited at 488 nm, images in the right column (**c**, **f**, **i**, **l**) are overlays of the Cy5 and PFS emissions. Scale bar is 8 μm. See online version for colored images

**Fig. 4:**
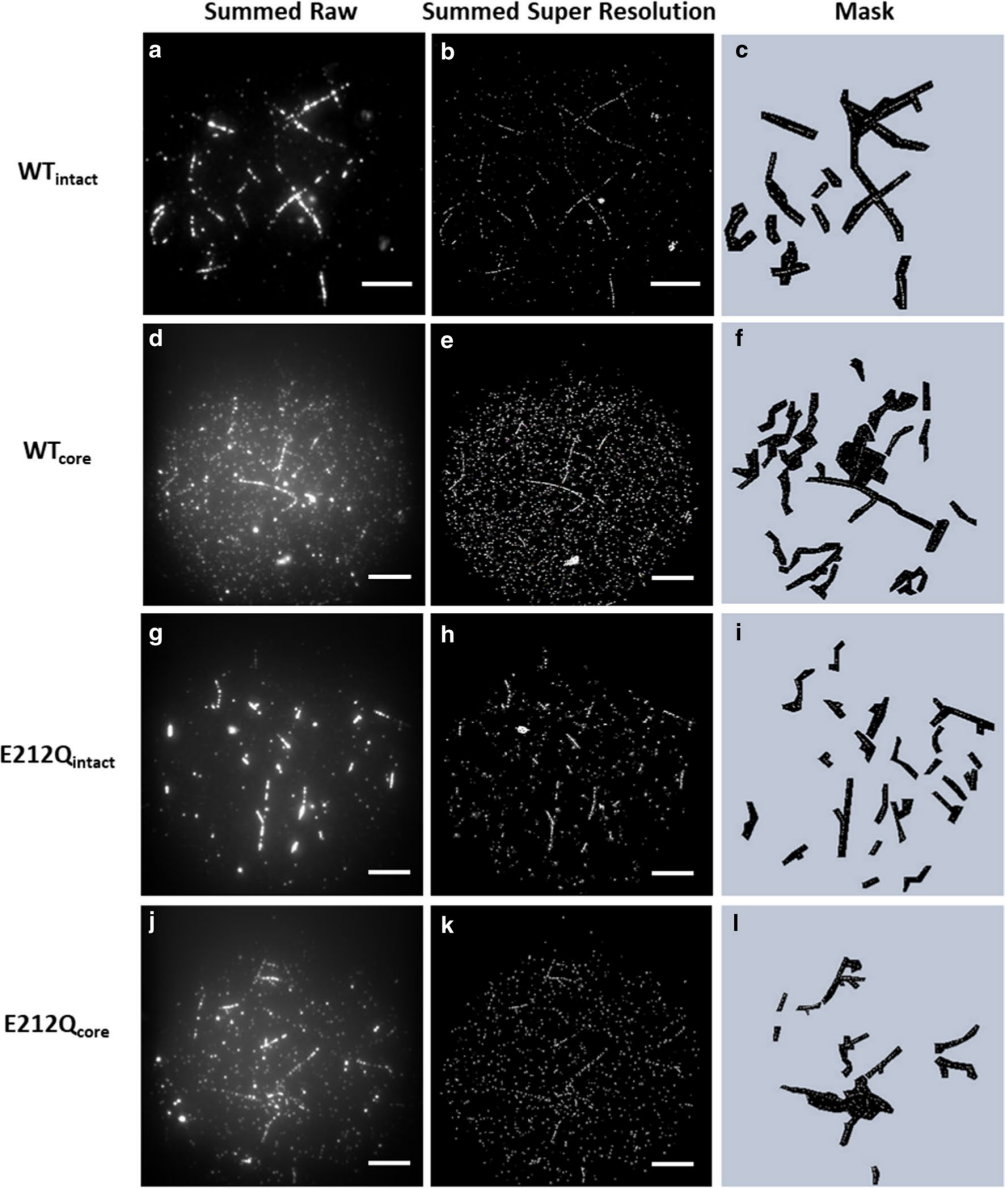
Image sequences (movies) consisting of 2500 frames were summed to determine the locations of fibrils. Summed image of raw data (**a**, **d**, **g**, **j**); summed image of the super-resolution image (**b**, **e**, **h**, **k**); masks were drawn around the fibrils to analyze binding of enzymes to cellulose. Spots in the dark regions in **c**, **f**, **i**, **l** were analyzed to determine binding times of enzymes to cellulose. Scale bars indicate 10 µm

WT_intact_ and E212Q_intact_ bound preferentially to cellulose rather than to the hydrophobic glass substrate (background), as visualized by colocalization of the emission from pontamine fast scarlet 4B (PFS) stained cellulose fibrils (Fig. 3b, h) and fluorescence from Cy5-labeled cellulases (Fig. 3a, g) in the overlays (Fig. 3c, i). In contrast, overlays of the corresponding core enzymes and the PFS-stained cellulose showed an abundance of spots bound to the background (Fig. 3d–f, j–l), indicating that the proteolytically isolated WT_core_ and E212Q_core_ had less targeted binding to cellulose. Moreover, even in the absence of PFS, Cel7A and E212Q (WT_intact_ and E212Q_intact_, respectively) concentrated immediately and specifically to cellulose such that traces of the fibrils were easily identifiable from binding patterns of the fluorescently labeled enzymes in each frame (e.g., Fig. 1b and Additional file 2). For CBM-less core versions of these enzymes (WT_core_ and E212Q_core_), fibrils were less apparent despite a tenfold increase in enzyme loading, and in some cases only identifiable when all 2500 frames of images were summed (e.g., Fig. 4d, e, j, k). The non-specificity of CD binding to cellulose has been reported previously and attributed to the lack of the carbohydrate-binding domain implicated in targeting cellulases to the cellulose surface [25]. Here, we observe that Cel7A without the linker and CBM bind readily and abundantly to the hydrophobic glass surface despite passivation with BSA. Similar results were obtained when PASC was used as the cellulose substrate, shown in Additional file 1: S6.

### Cel7A and Cel7A E212Q exhibit short and long residence times on cellulose

The interaction of WT_intact_ with cellulose fibrils appeared relatively stationary, with many enzymes residing on the cellulose fibrils for long periods (Additional file 2). Some enzymes bound and unbound during the window of observation; in areas that do not appear to have fibrils, enzymes appeared and disappeared rapidly. WT_core_ coverage on the imaging surfaces was more distributed and more dynamic than WT_intact_ (Additional files 3, 4, 5). There were some WT_core_ enzymes that remained for long durations on the surface, but it was less clear if these are bound to cellulose because they did not obviously align as if on a fibril. It is possible that some of these distributed enzymes are bound to the nanoparticles on the surfaces (Fig. 2a). E212Q_intact_ binding to cellulose appeared largely stationary (Additional files 6 and 7), while E212Q_core_ at the imaging surface were more spatially distributed and dynamic than E212Q_intact_ (Additional files 8 and 9). In general, cellulose fibrils were easily traced in experiments with WT_intact_ (Additional file 2) and E212Q_intact_ (Additional files 6 and 7) as the fluorescently labeled enzymes bound along the length of the fibrils. In contrast, because of the sparser binding of WT_core_ (Additional files 3, 4 and 5) and E212Q_core_ (Additional files 8 and 9), enzyme loading had to be increased to be able to trace the cellulose fibrils.

**Fig. 5:**
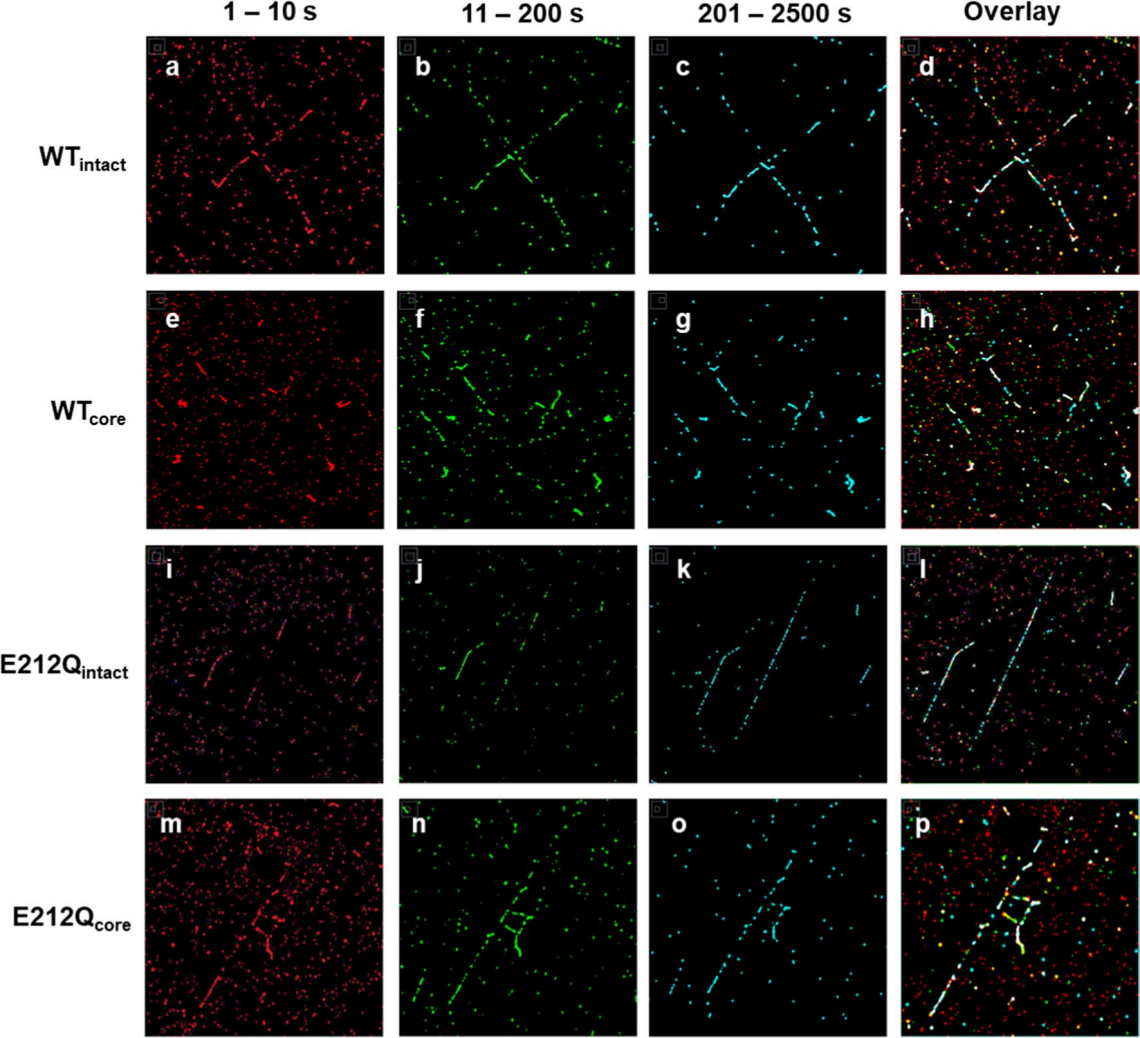
Super-resolution summed images of enzymes separated into residence time ranges of 1–10 s (red; **a**, **e**, **i**, **m**), 11–200 s (green; **b**, **f**, **j**, **n**), 201–2500 s (cyan; **c**, **g**, **k**, **o**). Overlay images (**d**, **h**, **l**, **p**) are constructed from overlays of the three. Image sizes are ~ 20 × 20 µm^2^. The image contrasts were enhanced to aid visualization

Super-resolution reconstructions of the raw image sequences pinpointed the locations of the bound enzymes, allowing us to determine the residence times of each enzyme bound to cellulose. Separating the super-resolution summed images (i.e., a combined image of all collected frames) into three binding time ranges (< 10 s, 11–200 s, 201–2500 s) revealed a general tendency of all four Cel7A variants to have short residence times (< 10 s) on the background and longer residence times on cellulose fibrils (Fig. 5). Again, even though the hydrophobized glass surfaces were treated with BSA prior to the addition of the enzymes in the imaging channels, Figs. 3 and 5 indicate that the passivation did not necessarily prevent enzyme binding to the background. However, in Fig. 5 we see that the enzymes do not accumulate on the background; the enzymes touch down on the surface, but leave shortly thereafter. The traces of the fibrils are increasingly pronounced with longer residence time ranges, indicating that enzymes that bind to cellulose tend to remain bound longer than 10 s.

**Fig. 6:**
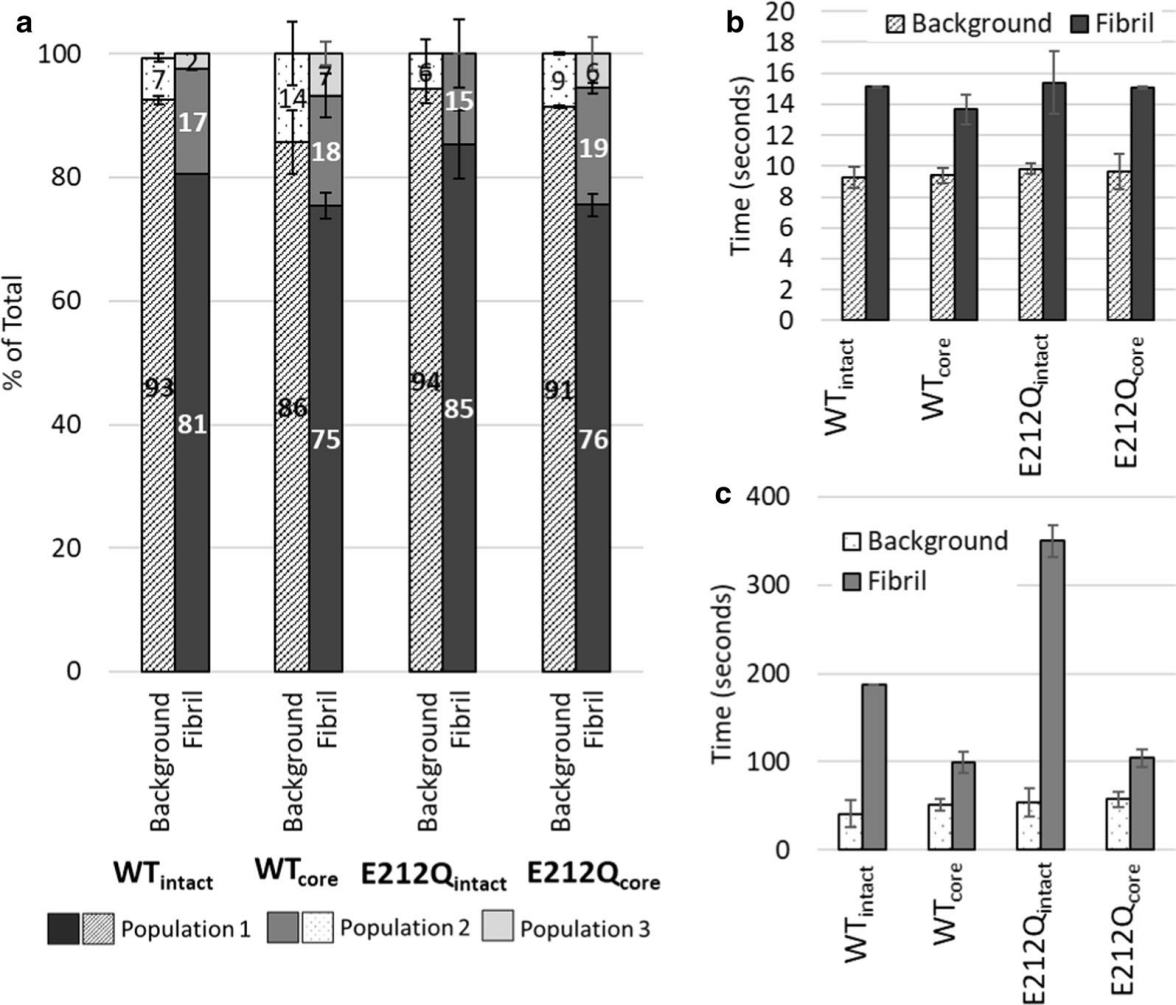
**a** Partitioning of Cel7A variants bound to the background and on the cellulose fibrils in 2–3 populations with distinct characteristic residence times; **b** characteristic residence times of Population 1 on the background and on the fibrils; **c** characteristic residence times of Population 2 on the background and on the fibrils. Characteristic residence times of Population 3 on the cellulose fibrils for WT_intact_, WT_core_, and E212Q_core_ were 1260 s, 389 ± 44 s, and 645 ± 225 s, respectively. Error bars represent standard deviations from three independent data sets or the spread between parameter estimates from two independent data sets. Fitting parameters for all data sets are provided in Table S2 of Additional file 1

To determine characteristic binding times of Cel7A on cellulose, the binding times of the enzymes that bound and unbound from cellulose fibrils during the observation window were compiled into histograms. The binding time histograms of the Cel7A variants were best fit by two- or three-exponential decays, indicating 2–3 populations differing in characteristic binding times interacting with cellulose (Fig. 6). Most of the enzymes bound to cellulose (75–85%) had short residence times of 14–15 s (Population 1 in Fig. 6a, b). Similar analysis of binding to the background (where no cellulose was present) also indicated short residence times by the majority (~ 90%) of the enzymes (Fig. 6a, b), which is consistent with our observations in Fig. 5d, h, l, p. The characteristic residence time of Population 1 Cel7A on the fibrils (14–15 s), however, was longer than on the background (~ 10 s), indicating an enhanced affinity of this enzyme to cellulose. Moreover, this enhanced affinity to cellulose relative to hydrophobized glass is not attributable to the CBM as the core versions of the enzymes behaved similarly.

As expected from the trends in Fig. 5, Cel7A enzymes bound for prolonged durations on cellulose. WT_intact_ appeared to have extended binding times of 187 s (Population 2) and 1260 s (Population 3) (Fig. 6a, c). However, these times reflect the photobleaching times of the Cy5 dye of 195 s and 1100 s (Additional file 1: S2). Thus, the analysis of WT_intact_ residence times on cellulose was subject to photo-physical limitations of the Cy5 label; i.e., ~ 20% of the WT_intact_ were effectively immobilized on the cellulose fibrils. Therefore, rather than 3 populations, we can only consider 2 bound populations of WT_intact_ bound to cellulose—a short-lived, but majority (81%) population of 15 s, and a minority (19%) population that is immobilized. Binding analysis of E212Q_intact_ also suggests a long-lived population (Population 2) bound to cellulose (Fig. 6c) for longer than the characteristic time for the Cy5 dye to photobleach. In the case of E212Q_intact_, the long-lived component was not resolved into two populations because of a lower number of enzymes included in the analysis. Nevertheless, we see that 15% of this enzyme was effectively immobilized on cellulose.

The core versions of Cel7A and E212Q, without linker and CBM, exhibited a population with prolonged binding to cellulose not truncated by photobleaching of the Cy5 dye (Fig. 6c); binding times of population 2 of WT_core_ and E212Q_core_ were 99 ± 12 s and 104 ± 10 s, respectively. Both enzymes also appeared to have a small fraction (6–7%) that bound for > 200 s (Population 3). Taken together, of the population of WT_core_ and E212Q_core-_ that bound for extended durations, ~ 75% released within ~ 100 s while ~ 25% remained immobilized. This was in contrast to the intact enzymes where 100% of the bound enzyme with extended binding times appeared to be immobilized.

## Discussion

### Immobilized Cel7A on cellulose

**Fig. 7:**
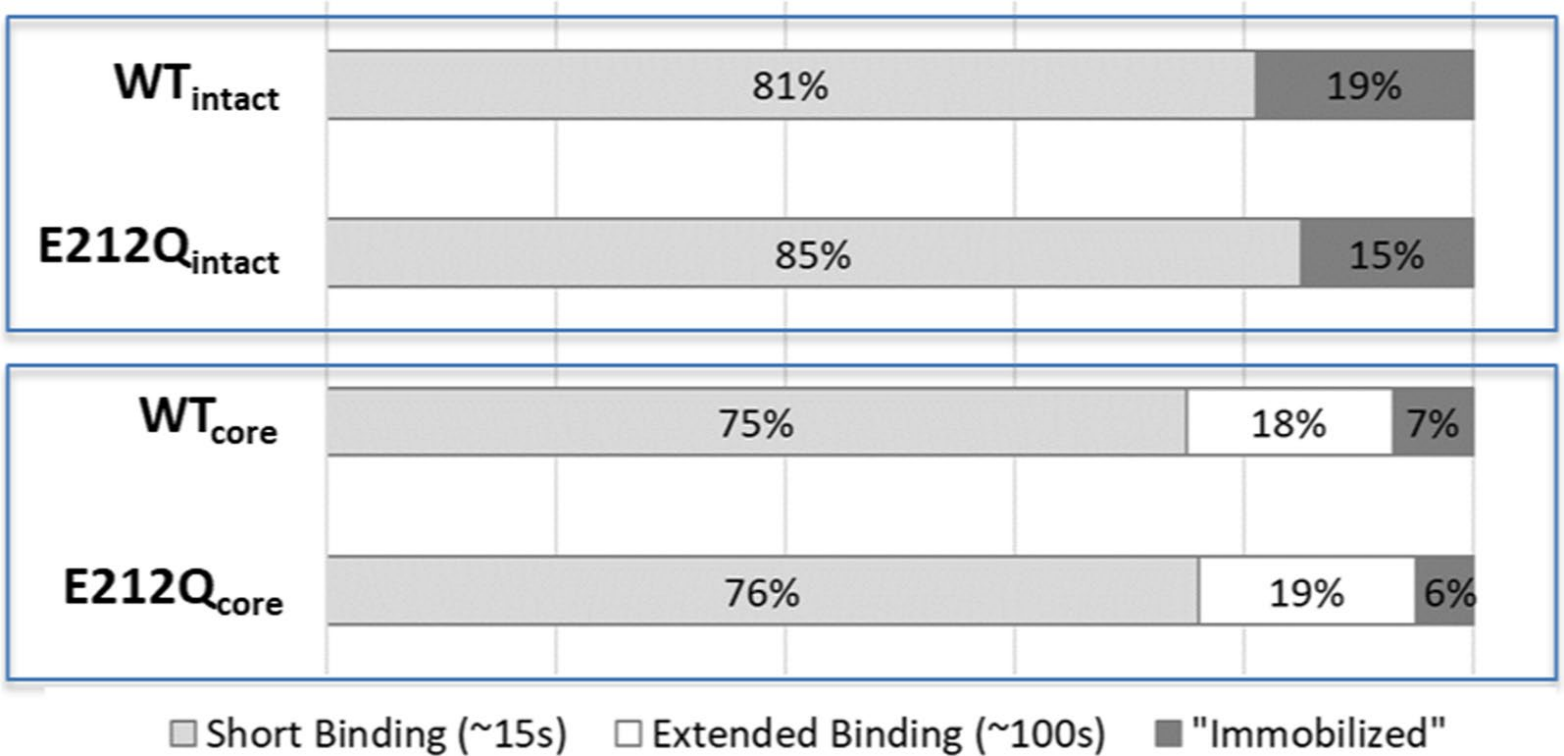
The majority (> 80%) of intact Cel7A (WT_intact-_ or E212Q_intact_) exhibited short binding lifetimes on cellulose fibrils, with < 20% appearing to be effectively ‘immobilized’. A large fraction (~ 75%) of the Cel7A catalytic domains (WT_core_ and E212Q_core_) also exhibited short binding lifetimes. Approximately 20% of the core enzyme bound for ~ 100 s, with only 6–7% ‘immobilized’

All the Cel7A variants exhibited a small, but significant cellulose-bound population with binding times exceeding the limit of photostability of the Cy5 fluorophore (Figs. 6, 7). In our hands, even with the oxygen-scavenging buffer and further decreases in laser intensity to extend fluorophore lifetimes, we were unable to determine the upper limit of the binding times. Additionally, in all the data sets, there were always several enzymes (~ 0.1–5%) that were bound from the first to the last frame (Additional files 2, 3, 4, 5, 6, 7, 8, 9) [26]. In a data set of 2500 frames, the binding durations of those enzymes exceeded 41.7 min. Hence, we have come to refer to these long-lived enzymes on cellulose as ‘immobilized’ enzymes. Taking the photostability of the Cy5 dye into account, distinct binding behaviors differentiating the intact Cel7A enzymes (WT_intact_ and E212Q_intact_) and the truncated catalytic domains of these enzymes (WT_core_ and E212Q_core_) emerge (Fig. 7). Intact Cel7A exhibited two types of binding—short-lived (< 15 s) and ‘immobilized’, while the cores exhibited three types of binding—short-lived (< 15 s), extended binding (~ 100 s) and immobilized. We speculate that these immobilized Cel7A are those that are complexed but inactive (i.e., not carrying out hydrolysis) at the cellulose interface.

**Fig. 8:**
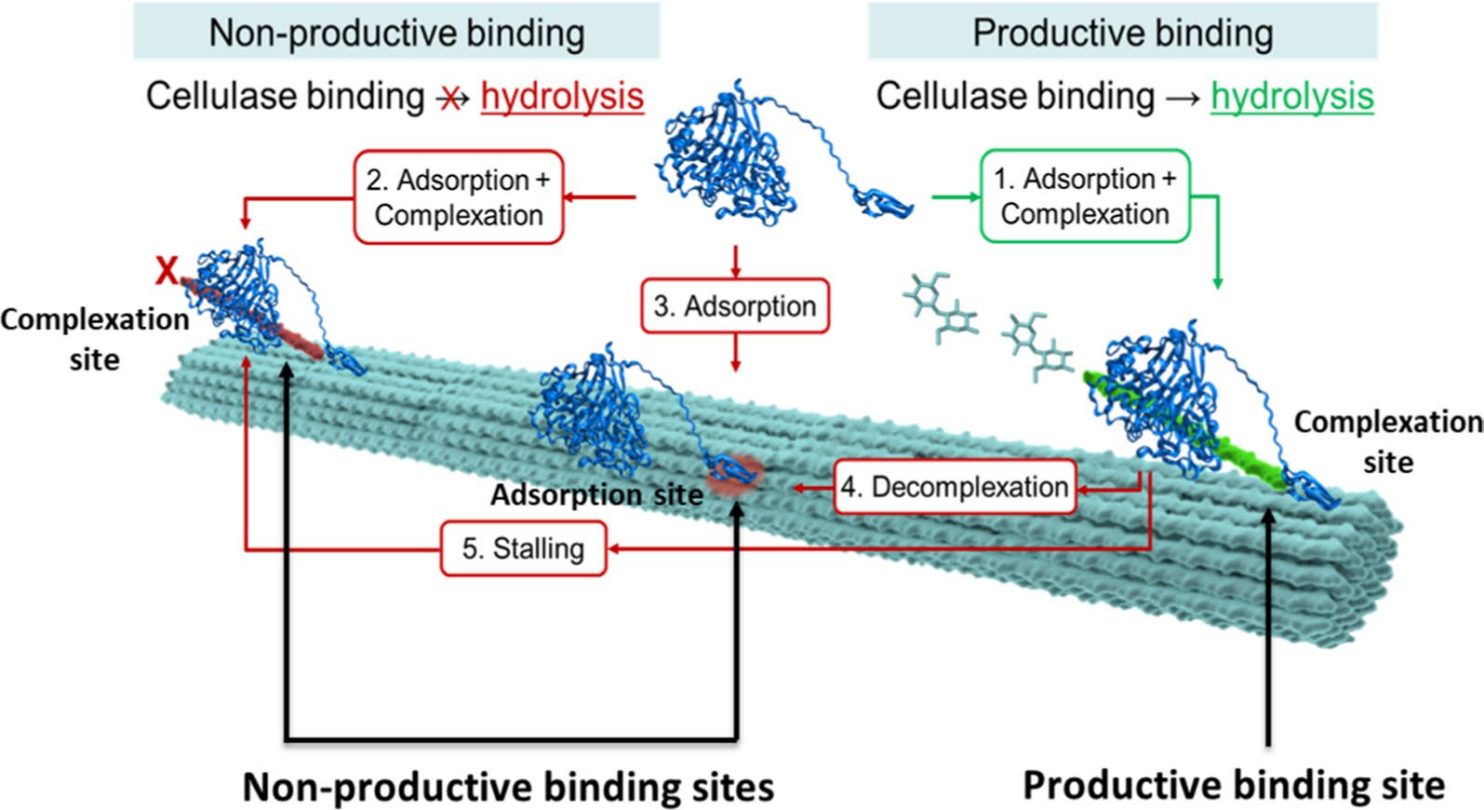
Schematic of Cel7A–cellulose interfacial interactions leading to non-productive and productive binding. Sites at which Cel7A fully loads its active site with a cellodextrin chain are complexation sites while sites at which Cel7A binds without engaging its active site (e.g., only by its CBM) are adsorption sites. Productive binding occurs at complexation sites where complexed enzymes hydrolyze cellulose; non-productive binding occur at adsorption sites and at complexation sites where complexed enzymes cannot carry out hydrolysis (illustration adapted from [13])

The interaction of Cel7A with cellulose can be parsed into the following elementary steps—adsorption/desorption, complexation/decomplexation and hydrolysis [6]. Correspondingly, the binding sites available for Cel7A at the substrate interface are complexation sites and adsorption sites (Fig. 8). Complexation sites are those at which Cel7A can fully engage with a cellodextrin within its active site. A complexed Cel7A actively hydrolyzing cellulose is considered to be a productively bound Cel7A (i.e., producing product) [23]. A complexed Cel7A that is not actively hydrolyzing cellulose is non-productively bound. Thus, as illustrated in Fig. 8, Cel7A bound to a complexation site can be either productive or non-productively bound, depending on whether it is actively hydrolyzing cellulose. Adsorption sites are sites at which Cel7A bind to cellulose without engaging its catalytic domain (e.g., binding only by the CBM).

We recently demonstrated that the productive cellulase binding capacity, i.e., the number of productive binding sites per mass of cellulose (moles/g) limits hydrolysis rates of cellulose [14, 23]. Nill and Jeoh [13] further found that during cellulose hydrolysis by purified Cel7A, a fraction of the productive binding sites on cellulose become unavailable due to complexed but inactive (i.e., non-productively bound) Cel7A that persist on the substrate. The algal cellulose used in the current study had an initial productive binding capacity of 0.83 µmol/g. While the extent to which Cel7A blocks productive binding sites appears to be cellulose source dependent, we found that at an enzyme loading of 5 µmol/g at 50 °C, WT_intact_ blocked 25 ± 5% of the productive binding sites on this cellulose. The immobilized fractions of WT_intact_ and E212Q_intact_ observed in the TIRFM experiments were 19% and 15% of the bound enzymes, respectively.

The evidence for a fraction of blocked productive binding sites on cellulose and the evidence for an immobilized fraction of bound enzymes on cellulose together provide a case for an obstructive tendency of Cel7A at the cellulose interface. Nill and Jeoh [13] demonstrated that obstruction of the productive binding sites by irreversibly bound Cel7A contributes to the premature decrease of overall hydrolysis rates. Our data comparing WT and E212Q show that Cel7A’s tendency to become immobilized at the cellulose interface does not depend on the enzyme’s ability to hydrolyze cellulose. Rather, the extensive binding interactions in the active site tunnel of Cel7A dictate binding times [27–29]. Why Cel7A becomes immobilized on the cellulose surface is still unclear and some have speculated that surface ‘obstacles’ limit processivity and prevent desorption [16]. Further, the question remains if this phenomenon is a consequence of the absence of accessory enzymes such as endoglucanases and lytic polysaccharide monooxygenases (LPMOs) typically co-expressed and synergistic with Cel7A.

### Catalytic domains have lowered affinity and find fewer complexation sites on cellulose

Without the CBM and linker, WT_core_ and E212Q_core_ exhibited reduced specificity to cellulose, reduced residence times and reduced size of the immobilized fraction on cellulose fibrils. The CBM of Cel7A has long been shown to increase the affinity of the enzyme to cellulose [21] and recently calculated to contribute − 5.4 kJ/mol to the standard free energy of Cel7A binding to cellulose [29]. The glycosylated linker joining the CBM and the CD of Cel7A has also been shown to have affinity to cellulose [10]. Further, studies suggest that the linker of Cel7A is optimized such that modifications of length or glycosylation on the linker peptide generally decreased the affinity of Cel7A to cellulose [30]. Thus clearly, the CBM and linker have the overall impact of increasing Cel7A’s affinity for cellulose.

We additionally observed sparser coverage of WT_core_ and E212Q_core_ on the cellulose fibrils (Additional files 3, 4, 5, 8, 9) [26]. Although the explanation may simply be that the reduced affinity from the lack of CBM results in reduced cellulose fibril coverage by the enzymes, the similarity in the short-lived binding time of ~ 15 s for the majority population of all the Cel7A variants (with and without CBM) suggests otherwise. Several studies have demonstrated biochemically that the isolated catalytic domain of Cel7A accesses fewer productive binding sites on cellulose [31–33]. Further, the CBM and linker have been shown to participate in the recognition and complexation of the enzyme to cellulose [34, 35]. Thus a more refined explanation for sparser coverage of WT_core_ and E212Q_core_ on cellulose is that without the CBM and linker, Cel7A recognizes and complexes to fewer sites on cellulose.

The fact that the intact enzymes had a larger immobilized fraction than the cores, and that the cores had a population with extended but shortened binding times imply that the CBM and linker contribute to the immobilization of Cel7A on cellulose. While the catalytic domain of Cel7A find fewer complexation sites on cellulose, productively bound Cel7ACD in fact have higher specific activity on cellulose [29, 36]. In interfacial kinetics such as that of cellulose hydrolysis, there is an optimum interplay between substrate affinity and specific activity to maximize overall activity of Cel7A [36]. Westh and coworkers describe the activity of intact Cel7A as desorption-limited and that of the Cel7A catalytic domain as adsorption-limited. In other words, the advantage in finding and complexing to sites on cellulose conferred by the CBM can come at a cost of preventing/slowing dissociation even after the enzyme is no longer actively hydrolyzing; conversely the absence of CBM reduces opportunities to hydrolyze cellulose but also does not prolong unproductive binding of Cel7A.

### How long do catalytically active Cel7A spend on cellulose?

The original motivation for this study was to visualize processive Cel7A on cellulose fibrils and to answer the question of how long catalytically active Cel7A enzymes remain bound to cellulose. The single-molecule experiments track the time spent by every enzyme in each field of view from the time it first appears to the time it desorbs and disappears from the surface. The appearance of the enzyme in the field of view indicates that, at a minimum, it has adsorbed to the surface. During the time that this enzyme is observed in the image sequence, it could undergo complexation, hydrolysis, decomplexation, and desorption from the surface of cellulose. Moreover, as illustrated in Fig. 8, binding duration can also include time spent complexed but inactive. Unfortunately, the current experimental setup cannot distinguish between productively and non-productively bound enzymes.

What we did find are the two binding populations of Cel7A on cellulose—one where most spend 15 s or less and another that is effectively immobilized. Nill and Jeoh [13] found that the immobilized Cel7A on cellulose were not productively bound; however, they also speculated that these are complexed Cel7A that were initially productive but became stalled and non-productive without decomplexing. A popular hypothesis speculates that processive hydrolysis of Cel7A can become stalled by physical obstructions on the surface of the substrate, but the residence time of complexed Cel7A is determined by the thermodynamics of the interactions between the catalytic site residues and the complexed cellodextrin [29]. Simply stated, residence times of complexed Cel7A are expected to be longer than the time during which they are actively hydrolyzing cellulose.

If the short-lived Cel7A population on cellulose were active throughout its binding time, potential supporting evidence is unidirectional movements of Cel7A along the surface of cellulose fibrils due to processive hydrolysis. The intrinsic processivity of Cel7A actively hydrolyzing cellulose can be estimated as a ratio of its turnover number and dissociation rate constant [16]. The turnover number, or catalytic rate constant (k_cat_), and the dissociation rate constant of *T. reesei* Cel7A, have been reported in the ranges of 2–11 s^−1^ and 0.14–0.0007 s^−1^, respectively [14]. The characteristic residence time of the short-lived bound Cel7A population of 15 s corresponds to a dissociation rate of 0.067 s^−1^, well within the range of the previously reported rates. Using the definition above, the intrinsic processivity of the short-lived Cel7A on cellulose in these experiments could range between ~ 30 and 165 turnovers. Cel7A has been reported to processively hydrolyze cellulose for ~ 15–90 consecutive catalytic cycles each time it binds productively to cellulose [14]. Experimentally determined processivities have been viewed as being truncated with respect to the intrinsic processivity of Cel7A and strongly substrate dependent. Interestingly, our estimated intrinsic processivity falls in the general range of the experimental values. Given that each turnover moves the enzyme ~ 1 nm [37], the short-lived bound population of Cel7A could translate ~ 30–165 nm per hydrolytic run. Most of the bound enzymes in the raw data do not appear to translate (Additional files 2, 3, 4, 5, 6, 7, 8, 9) [26], which may simply be due to a lateral resolution of 220 nm/pixel. Even with super-resolution localization of the centroids of each spot in the image sequences, detection of unambiguous sliding movements of enzymes was exceedingly difficult. There were occasional observations of enzyme movements (e.g., Additional file 10), but these events were rare. The raw data sets with accompanying super-resolution centroid coordinates are published and available for additional analyses by others [26].

We must note that E212Q, being catalytically deficient and unable to processively hydrolyze cellulose exhibited the same binding times as the WT (Fig. 6). E212Q also had a short-lived binding population with a characteristic binding time of ~ 15 s and an ‘immobilized’ population on cellulose. Moreover, as already discussed, the catalytic domains of these two enzymes also exhibited the ~ 15 s characteristic residence time. One could take the common residence times of the Cel7A variants to posit that dissociation of Cel7A is limited by the disengagement of its catalytic domain from the complexed cellodextrin. Decomplexation of Cel7A requires ‘dethreading’ of the cellodextrin chain from the active site tunnel, requiring the extraction of ~ 8 glucose residues forming the equivalent number of interactions in the tunnel [28]. Our data certainly suggest that dissociation is not limited by hydrolytic activity. Thus, we return to the conclusion that while it is possible that Cel7A is active throughout its residence on cellulose, it certainly does not have to be.

### Additional evidence for Cel7A activity in the single-molecule imaging experiments

**Fig. 9:**
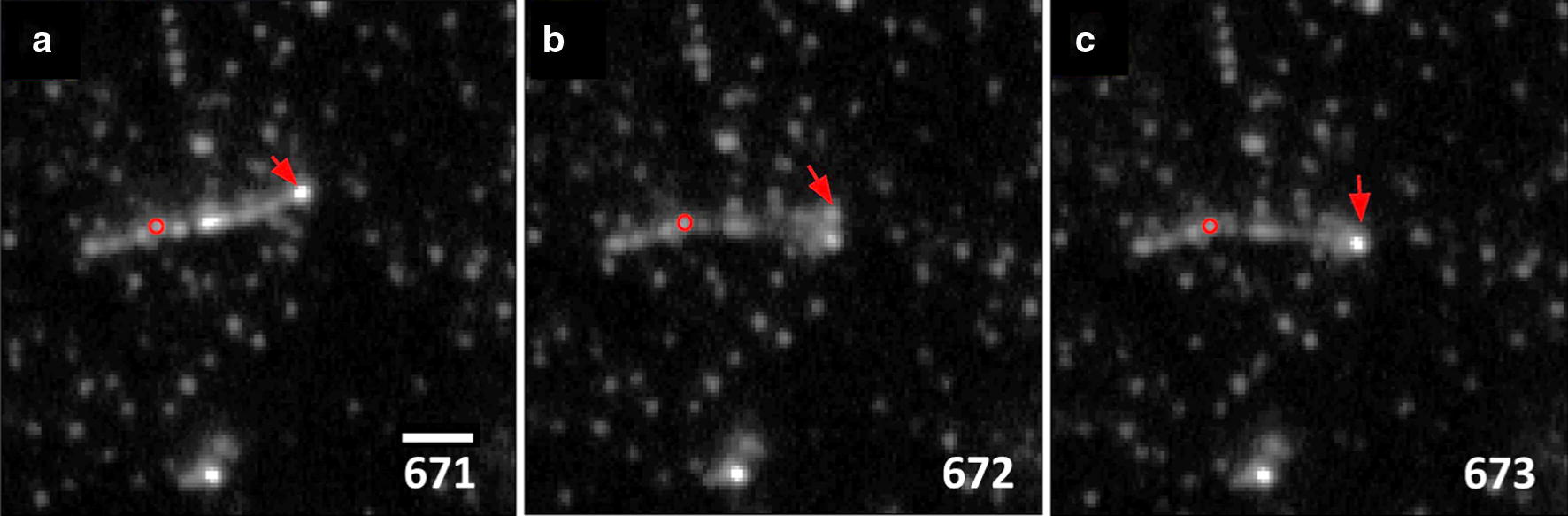
Three successive frames (1 frame/s) (**a**–**c**) that capture formation of a kink in an algal cellulose fibril treated with WT_core_. Red arrows point to the fibril tips while the red circle shows the pivot point about which the fibril kinks. Frame numbers are shown in each panel. Video sequence for this and a second observation are shown in Additional files 11 and 12. Scale bar indicates 2 µm

In two separate instances during the course of observing enzyme binding to cellulose, fibrils with associated enzymes were seen to kink along the lengths (frames—671–673 in Fig. 9a–c and Additional file 11; frames 234–236 in Additional file 12). Earlier works have shown that as Cel7A hydrolyze cellulose, the microstructure undergoes structural changes such as fibrillation and segmentation [38, 39]. Moreover, computational modeling based on transmission electron microscopy revealed that cellulose microfibrils kink along their lengths in their energy minimized state [40]. Ciesielski et al. proposed that highly crystalline cellulose fibrils kink to release internal stresses [41]; we speculate that we observed this stress release due to the cellulose fibrils being actively hydrolyzed. Although the process of kinking was only observed twice, kinked fibrils were frequently observed in the experimental samples.

## Conclusions

We have measured the binding lifetimes of Cel7A, its catalytically deficient mutant, E212Q, and their respective catalytic domains on cellulose. All the Cel7A variants bind non-specifically to hydrophobized glass, even when passivated by BSA; non-specific binding, however, is highly dynamic with characteristic binding times < 10 s. All the Cel7A variants were more specific and had stronger affinity to cellulose than to the hydrophobized glass, regardless of whether the variant had a CBM and linker. The lack of CBM and linker, however, manifested clearly as reduced concentration of the enzyme bound to cellulose. From our observations, we speculate that the CBM and linker increases the affinity of Cel7A to cellulose possibly by facilitating recognition of complexation sites and by aiding in the uptake of a cellodextrin chain into the enzyme active site. The increase in the affinity of the intact Cel7A due to the CBM and linker also appeared to come at the cost of increasing the fraction of immobilized enzymes on cellulose. Removing the CBM and linker resulted in a fraction of the long-lived bound Cel7A catalytic domain population to dissociate within ~ 100 s. The wild-type and catalytic-deficient Cel7A catalytic domains exhibited similar binding behavior on cellulose, thus supporting that residence time (or conversely dissociation rates) of Cel7A from cellulose is not catalysis limited. The crystalline cellulose fibrils used in this study were highly polished to reduce the number of productive Cel7A binding sites. Consequently, the cellulose surface coverage of bound Cel7A was vastly reduced. Binding behavior between intact and truncated core versions of Cel7A was similar. Taken together, we conclude that the Cel7A–cellulose interactions measured in our study were primarily bound by the catalytic domain. The cellulose-binding module facilitates the catalytic domain, but does not appear to be the driver for interactions of Cel7A at the interface of cellulose.

## Methods

### Cellulose preparation

Algal cellulose (AC) was purified from *Cladophora aegagropila* by sequential and repeated alkali and acidified hypochlorite treatments as described previously [23, 42]. Isolated algal cellulose fibrils were further treated with 5 M hydrochloric acid at 70 °C overnight [13]. The residual AC fibrils were washed thoroughly with water to remove excess acid, then stored at 4 °C with 0.02% sodium azide until further use. The productive Cel7A binding capacity of the AC was determined from initial cellobiose production rates at saturating conditions as previously described [13, 23].

### Enzyme preparation

*Trichoderma reesei* Cel7A (WT_intact_) was purified from a commercial *Trichoderma reesei* cellulase preparation (Sigma Aldrich Catalog number C2720) as previously described [39, 43].

The E212Q gene was constructed with the p*GEM*-*T* easy vector (Promega Corporation, WI, USA) and transformed into *Escherichia coli* DH5α+ Amp^+^. The gene was extracted and ligated to the p*TrEno* vector with the *eno* promoter [44]. The *p*TrEno/E212Q vector, containing *eno* promoter and an E212Q fragment, was transformed into the *T. reesei* strain AST1116 via electroporation [45]. The *p*Treno plasmid expressed the E212Q in *T. reesei* in glucose-rich media without contamination from the CBHI wild-type. The transformed *T. reesei* AST1116 was spread onto potato-dextrose agar plates with hygromycin B as a selection marker at 30 °C until the sporulating lawn was observed. The colonies from the sporulating lawns were transferred to Mandels and Andreotti Medium (MA) with 1 M glucose and hygromycin B at 30 °C with 200 rpm and incubated for 3 days. The E212Q enzyme (E212Q_intact_) was purified by fast protein liquid chromatography (FPLC) through a multistep process, the details of which can be found elsewhere [45]. Briefly, the protein was isolated using hydrophobic interaction chromatography followed by anion exchange chromatography using a Resource Q column, a second round of hydrophobic interaction chromatography using a Resource ISO column and finally size exclusion chromatography. The purified product was confirmed as E212Q by Western blot analysis and protein sequencing [45]. The loss of cellulolytic activity of E212Q compared to the wild-type Cel7A was confirmed on the recalcitrant algal cellulose and highly digestible phosphoric acid swollen cellulose (Additional file 1: S1).

**Fig. 10:**
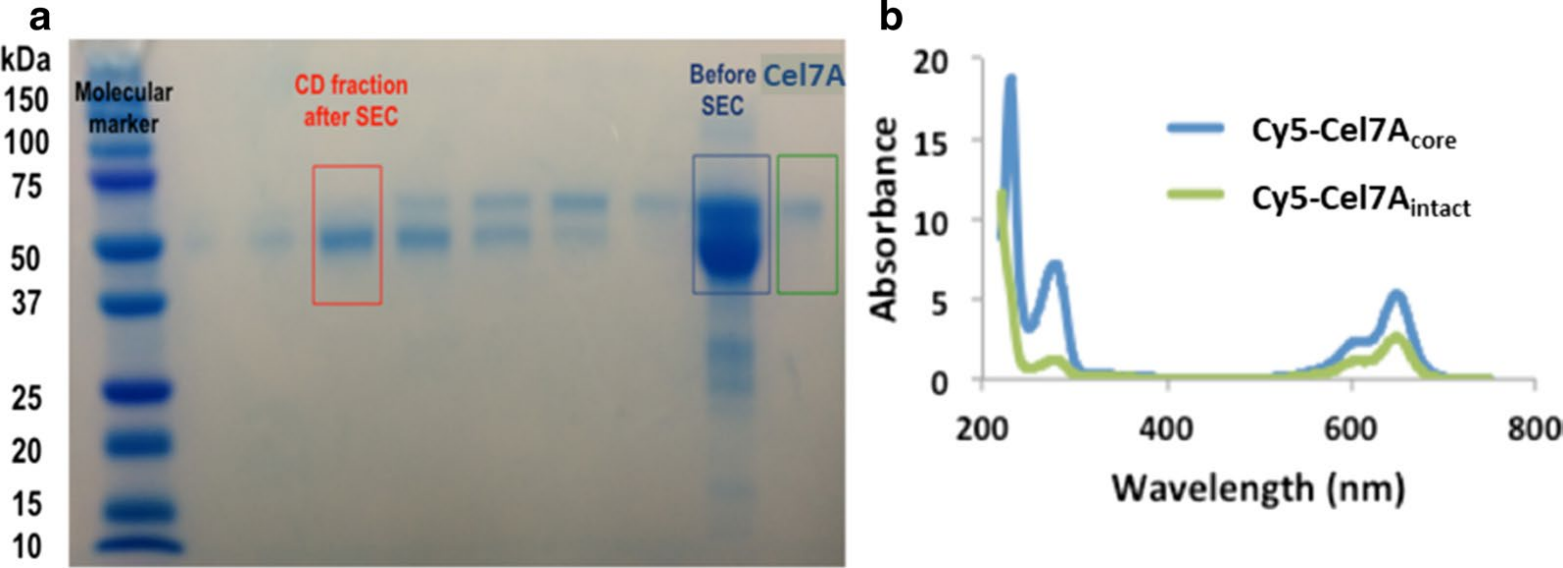
**a** SDS PAGE showing the different bands before and after size exclusion chromatography. **b** Absorbance spectra of Cy5-labeled WT_intact_ and WT_core_ enzymes

Isolated catalytic domains of Cel7A and E212Q (WT_core_ and E212Q_core_) were obtained by limited proteolysis of the purified Cel7A (WT_intact_) and E212Q (E212Q_intact_) [11, 46]. WT_intact_ or E212Q_intact_ was incubated with immobilized papain (Thermo Fisher Scientific, cat# 20341), equilibrated in the digestion buffer (20 mM sodium phosphate, 10 mM ethylenediamine tetraacetic acid, 20 mM cysteine HCl) for 7 h at 37 °C and gentle agitation. The supernatant containing the cleaved fractions were separated from the papain by centrifugation, then concentrated and separated by size exclusion chromatography (Superdex 200, GE healthcare) in 5 mM NaOAc and 100 mM NaCl, pH 5. The fractions containing the isolated catalytic domain as confirmed by SDS PAGE (Fig. 10) were pooled, concentrated, and stored for further use.

All enzymes were labeled with Cy5 fluorophore (GE Healthcare Life Sciences, Amersham) per manufacturer procedures and separated from excess dye using Zeba Desalting columns (Thermo Fisher Scientific) as previously described [17]. This procedure resulted in a degree of labeling (moles of Cy5/mole protein) of 0.71 and 0.25 for WT_intact_ and WT_core_, respectively, and 0.63 and 0.79 for E212Q_intact_ and E212Q_core_, respectively.

### Cellulose deposition on glass coverslips

Never-dried cellulose was deposited onto hydrophobically silanized glass coverslips by gravity-aided settling in the imaging channels. Imaging channels of 10 μL working volume were built onto the hydrophobized glass coverslips as described previously [17]. The channels were filled with cellulose suspensions (0.025–0.25 mg/mL), sealed, and allowed to settle until ready for use (minimum settling time was overnight in the refrigerator). Prior to use, unbound cellulose was rinsed off the surface with buffer and the channels were incubated with 10 mg/ml of bovine serum albumin (BSA) for > 10 min to passivate the surface. Successful deposition of cellulose fibrils onto the imaging surfaces was confirmed by AFM or by staining with pontamine fast scarlet 4B (PFS), a cellulose-specific fluorescent dye [47].

### Atomic force microscopy (AFM) imaging of cellulose

Imaging of cellulose adhered on hydrophobic glass coverslips was done in tapping mode in water using a MFP 3D Bio AFM (Asylum Research) with silicon AFM probes (AC240 TS, Asylum Research) as described earlier [39]. Scanning parameters were optimized for each acquisition. The images were processed using the Asylum Research MFP 3D template in Igor Pro (Wavemetrics, Inc.).

### Single-molecule fluorescence imaging

A through-objective total internal reflection excitation fluorescence microscopy (TIRFM) setup was used to collect single-molecule fluorescence images of individual Cy5-labeled enzyme molecules bound to cellulose fibrils. A detailed description of this setup is given elsewhere [48]. Briefly, 488 nm and 637 nm excitation laser beams were reflected by a multiline dichroic mirror (FF500/646-Di01, Semrock) and focused at the back aperture of a 1.49 NA 60× oil-immersion objective (Olympus) to provide total internal reflection (TIR) excitation at the cover glass/water interface across a ~ 50 µm diameter area in the object plane (Fig. 1a). Sample emission was collected and imaged by the same objective onto the 512 × 512-pixel sensor of an electron multiplying CCD (EMCCD) camera (Photonmax, Princeton Instruments). A 37-nm-wide bandpass filter centered at 676 nm was used to isolate Cy5 fluorescence excited at 637 nm. A 40 nm wide bandpass filter centered at 562 nm was used to isolate pontamine fast scarlet 4B (PFS) fluorescence excited at 488 nm. An image of Cy5-labeled Cel7A bound to cellulose collected in a field of view of 56 × 56 μm^2^ is shown in (Fig. 1b). For the binding time measurements, the overall magnification of the imaging system (73×) mapped each EMCCD pixel to a 220 × 220 nm area in the object plane. Moreover, relatively low power (~ 0.2 mW @ 637 nm) excitation was used to reduce the effect of Cy5 photobleaching on the binding time measurements. Image stacks were collected at EMCCD camera integration times of 1 s.

In a typical experiment, the Cy5-labeled enzymes in an oxygen-scavenging buffer at a concentration of 50–100 pM were loaded into the 10-μL channel and imaged over the course of 40–50 min at 1 frame per second to generate movies consisting of a series of sequential images (image stacks). The oxygen-scavenging buffer (glucose oxidase, catalase, 2% glucose and trolox in 50 mM sodium acetate buffer pH 5) was used to enhance Cy5 photostability and reduce fluorophore blinking [49]. Under the imaging conditions used in this study, immobilized Cy5 fluorophores exhibited two decay lifetimes of 195 s and 1100 s when illuminated in buffer with the oxygen-scavenging system. When the oxygen-scavenging buffer was not used, Cy5 decay lifetimes were 5 s and 20 s (see Figure S2 in Additional file 1). Compressed videos of the data used in this study are provided as Additional files 2, 3, 4, 5, 6, 7, 8, 9, 10, 11, 12 while the raw data are published and available elsewhere [26].

### Single-molecule image analysis to obtain enzyme-binding lifetimes

The image stacks were processed using Image J software (Version 1.49, NIH). The position coordinates of individual enzyme molecules adhered to cellulose in the image stacks were determined using DAOSTORM, a single-molecule fluorescence localization algorithm that was adapted from the astronomy software package DAOPHOT [22]. Detailed information about preparation of the raw datasets for analysis can be found in Additional file 1: S3. The output from DAOSTORM, a list of spot centroid *x*–*y* coordinates and fluorescence intensities of the individual localized enzyme molecules, was corrected for lateral drift of the microscope stage and analyzed with a custom algorithm developed in-house to count the number of frames during which individual enzyme molecules were present at given locations (*x*, *y*). The reconstructed super-resolution images generated from the DAOSTORM output were used to mask images to isolate binding time analyses on regions with (e.g., Fig. 4) or without fibrils. These data were used to compile binding time histograms that were fit to multiple exponential decay models using the differential evolution (genetic optimization) algorithm [50]. Enzymes found in the first and last frames of the image stack were not included as their total residence times could not be determined. The fits were minimized using Poisson deviance [51] as the cost function, implemented in Igor Pro (Wavemetrics, Version 6.34A) to determine characteristic binding times. The best fit was chosen based on the residual plots and reduced Poisson deviance value close to 1 to optimally fit the data (details on binding time analyses are provided in Additional file 1: S4 and S5).

A two-sample t test was used to determine significant differences between characteristic lifetimes of the binding populations. Separate statistical tests were conducted to compare each population for WT_intact_ and E212Q_intact_, WT_core_ and E212Q_core_.

## Supplementary information

**Additional file 1: S1.** Relative activities of Cel7A and E212Q used in this study. **S2.** Photostability of Cy5 labels under imaging conditions used in this study. **S3.** Localization of individual enzymes in a single-molecule (SM) fluorescence image sequence using DAOSTORM. **S4.** Obtaining Binding lifetimes of individual enzyme molecules using output from DAOSTORM. **S5.** Multi-exponential fitting of the binding time histograms. **S6.** Binding of Cel7A and Cel7A CD on phosphoric acid swollen cellulose (PASC). **S7.** Automating the analysis of single-molecule (SM) image stacks for unbiased determination of binding lifetimes of cellulases on cellulose.

**Additional file 2:** Cy5-labeled WT_intact_ in oxygen scavenging buffer with HCl treated algal cellulose fibrils. Data collected at 1 frame per second (fps). Playback at 20 fps. 56 µm × 56 µm. (TrCel7A111130.spe).

**Additional file 3:** Cy5-labeled WT_core_ in oxygen scavenging buffer with HCl treated algal cellulose fibrils. Data collected at 1 frame per second (fps). Playback at 20 fps. 66 µm × 66 µm. (Cel7ACD123032.spe).

**Additional file 4:** Cy5-labeled WT_core_ in oxygen scavenging buffer with HCl treated algal cellulose fibrils. Data collected at 1 frame per second (fps). Playback at 20 fps. 66 µm × 66 µm. (Cel7ACD133451.spe).

**Additional file 5:** Cy5-labeled WT_core_ in oxygen scavenging buffer with HCl treated algal cellulose fibrils. Data collected at 1 frame per second (fps). Playback at 20 fps. 66 µm × 66 µm. (Cel7ACD143742.spe).

**Additional file 6:** Cy5-labeled E212Q_intact_ in oxygen scavenging buffer with HCl treated algal cellulose fibrils. Data collected at 1 frame per second (fps). Playback at 20 fps. 66 µm × 66 µm. (E212Q160828).

**Additional file 7:** Cy5-labeled E212Q_intact_ in oxygen scavenging buffer with HCl treated algal cellulose fibrils. Data collected at 1 frame per second (fps). Playback at 20 fps. 66 µm × 66 µm. (E212Q170336).

**Additional file 8:** Cy5-labeled E212Q_core_ in oxygen scavenging buffer with HCl treated algal cellulose fibrils. Data collected at 1 frame per second (fps). Playback at 20 fps. 66 µm × 66 µm. (E212QCD181309.spe).

**Additional file 9:** Cy5-labeled E212Q_core_ in oxygen scavenging buffer with HCl treated algal cellulose fibrils. Data collected at 1 frame per second (fps). Playback at 20 fps. 66 µm × 66 µm. (E212QCD190922.spe).

**Additional file 10:** One example of lateral translation of Cy5-labeled WT_core_ along a cellulose fibril. Data collected at 1 fps, playback at 20 fps.

**Additional file 11:** Movement of the tip of an algal cellulose fibril treated with WT_core_ in 50 mM sodium acetate buffer, pH 5. The movement of the fibril tip can be observed between frames 671–673 shown by the red arrow. Data collected at 1 frame per second (fps). Playback at 10 fps, elapsed time 749 s. Scale bar is 2 μm.

**Additional file 12:** Movement of a fibril tip of algal cellulose treated with WT_core_ in 50 mM sodium acetate buffer, pH 5. The movement of the fibril tip can be observed between frames 234–236 shown by the red arrow. Data collected at 1 fps. Playback at 10 fps, elapsed time, 306 s. Scale bar is 1 μm.

## Data Availability

Additional data and compressed videos of the TIRFM image sequences supporting the conclusions of the article are included within the article and its additional files. Additionally, the datasets supporting the conclusions of this article are available in the University of California Dash data repository 10.25338/B8ZC80 [26]. Filenames of the published original raw data are given in the figure captions.

## Abbreviations

TIRFM: total internal reflection fluorescence microscopy
AFM: atomic force microscopy
AC: algal cellulose
PASC: phosphoric acid swollen cellulose
Cel7A: *Trichoderma reesei* Cel7A
