# Supplementary material for: Interfacial molecular interactions of cellobiohydrolase Cel7A and its variants on cellulose
Source: Biotechnol Biofuels. 2020 Jan 18;13:10. doi: 10.1186/s13068-020-1649-7 (PMC6969433; doi:10.1186/s13068-020-1649-7)
Supplement: Supplementary file 1 — Additional file 1: S1. Relative activities of Cel7A and E212Q used in this study. S2. Photostability of Cy5 labels under imaging conditions used in this study. S3. Localization of individual enzymes in a single-molecule (SM) fluorescence image sequence using DAOSTORM. S4. Obtaining Binding lifetimes of individual enzyme molecules using output from DAOSTORM. S5. Multi-exponential fitting of the binding time histograms. S6. Binding of Cel7A and Cel7A CD on phosphoric acid swollen cellulose (PASC). S7. Automating the analysis of single-molecule (SM) image stacks for unbiased determination of binding lifetimes of cellulases on cellulose. [file 13068_2020_1649_MOESM1_ESM.docx]

**Additional File 1**

Interfacial Molecular Interactions of Cellobiohydrolase Cel7A and Its Variants on Cellulose

^‡^Akshata R. Mudinoor, ^§^Peter M. Goodwin, ^¥^Raghavendra U. Rao, ^‡,†^Nardrapee Karuna, ^‡^Alex Hitomi, ^1,2^Jennifer Nill and *^‡^Tina Jeoh

^‡^Biological and Agricultural Engineering, University of California, One Shields Avenue, Davis, CA 95618

^§^Center for Integrated Nanotechnologies, Los Alamos National Laboratory, Los Alamos, New Mexico, 87545

^¥^Gracenote, Inc., 1900 Powell Street, Suite 2000, Emeryville, CA 94608

^†^Biotechnology, Faculty of Engineering and Industrial Technology, Silpakorn University, Nakhon Pathom, Thailand

^1^Chemical Engineering, University of California, Davis, One Shields Ave., Davis, CA 95616, United States

^2^Molecular Biophysics and Integrated Bioimaging, Lawrence Berkeley National Lab, 1 Cyclotron Road, Berkeley, CA 94720, United States

KEYWORDS: *Trichoderma reesei* Cel7A, super-resolution, single-molecule imaging, catalytic domain, binding lifetime, desorption rate, heterogeneous enzyme kinetics

## Relative activities of Cel7A and E212Q used in this study


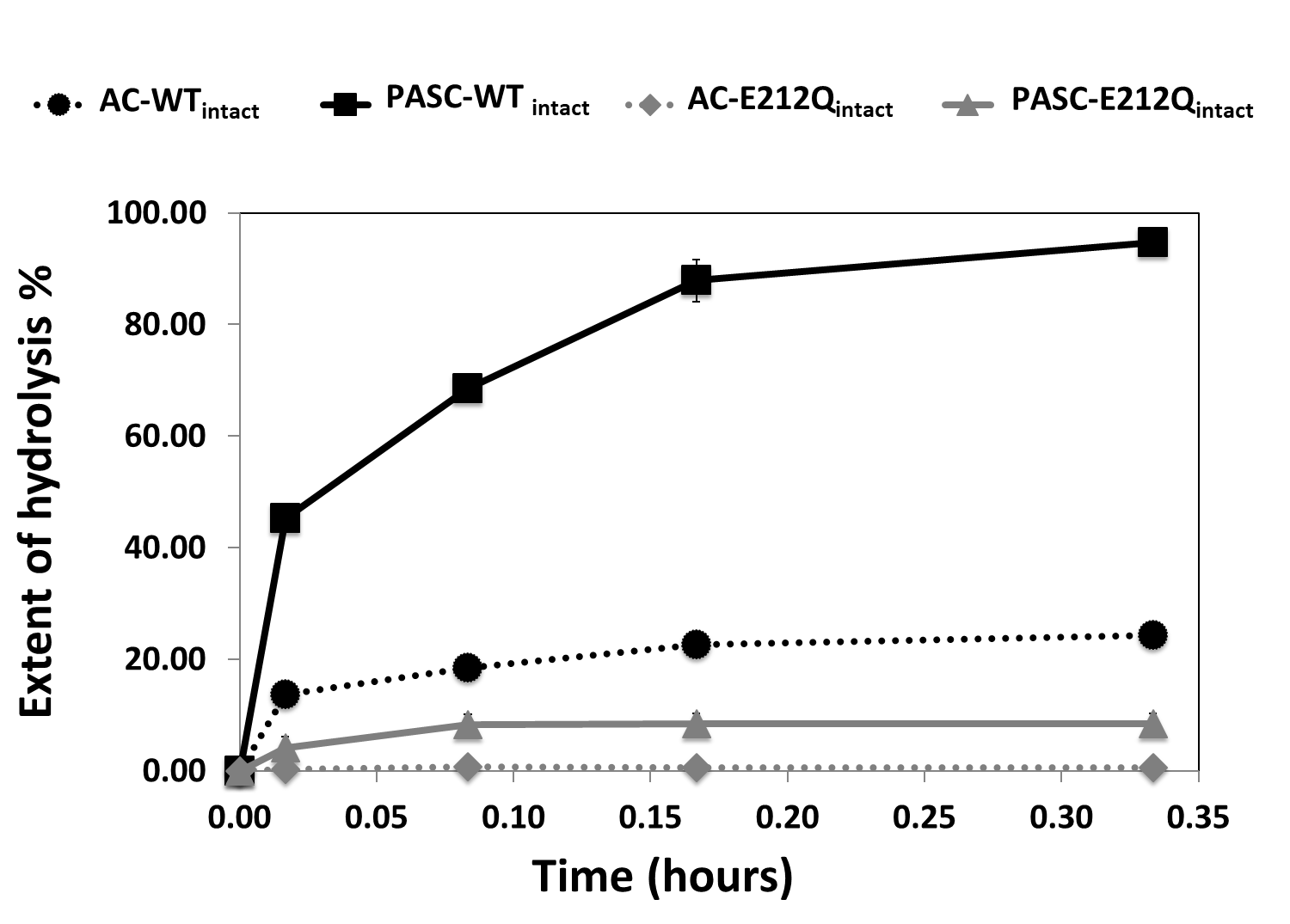
The single point E212Q mutation of Cel7A has been reported to diminish the enzyme activity on cellulose by 2000-fold (1). We confirmed significant loss in cellulolytic activity on both the recalcitrant algal cellulose (AC) and on the highly digestible phosphoric acid swollen cellulose (PASC) (Figure S.1).

Figure S.1. Hydrolysis time course of *AC and PASC*by WT_intact_*and*E212Q_intact_at 50°C at an enzyme loading of 10 μmoles/g cellulose. Error bars are the standard deviations of triplicate measurements and maybe obscured by the markers.

## Photostability of Cy5 labels under imaging conditions used in this study

Cy5-labeled streptavidin was bound to a biotinylated imaging surface and imaged with the TIRFM under the same experimental conditions detailed in the main manuscript. The fluorescence intensity was measured over time with and without the use of the glucose oxidase/catalase (GODCAT) oxygen scavenging buffer system (glucose oxidase, catalase, 2% glucose and trolox in 50 mM sodium acetate buffer pH 5) (2). The fluorescence intensity decays under both conditions were well-described by a biexponential decay (Figure S.2). The oxygen scavenging buffer system clearly extended the photo-stability of the Cy5 dye as shown in Table S.1.

##
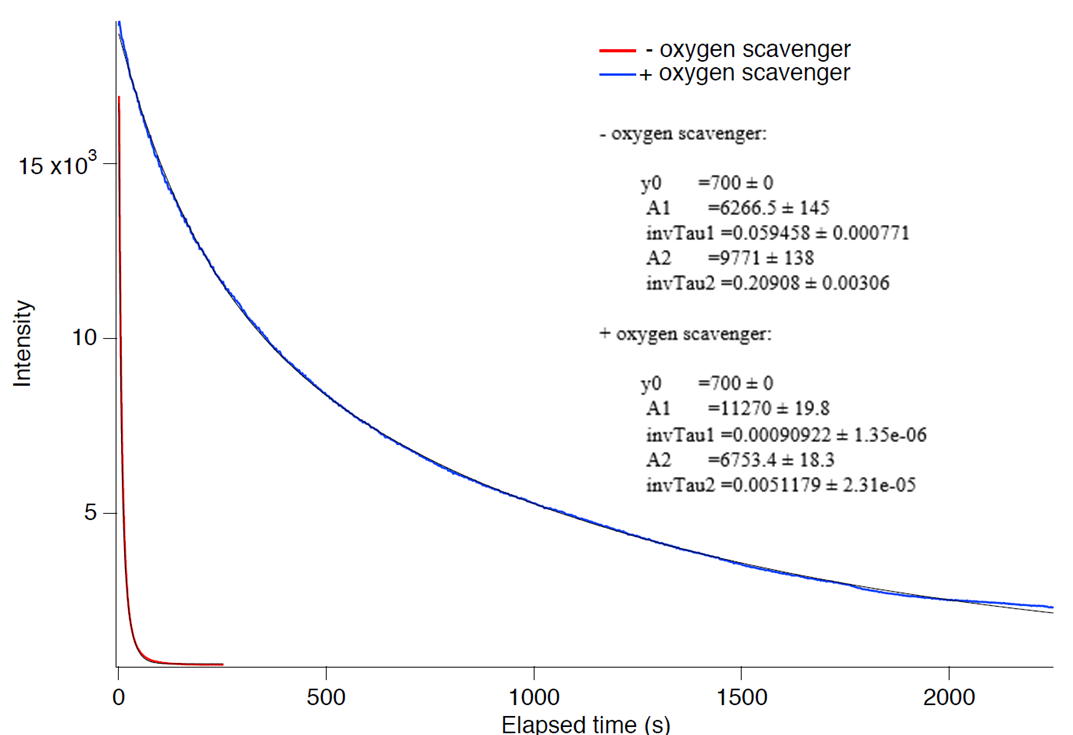


Figure S.2. Fluorescence intensity of Cy5-streptavidin immobilized on a biotinylated imaging surface measured with and without the oxygen scavenging buffer. The data with oxygen scavenger are shown in blue while the data without oxygen scavenger are shown in red. The fits to the data are in black for both data sets.

Table S.1: Fluorescence lifetimes of Cy5-labels with and without the use of the oxygen scavenging buffer system.

|  | POPULATION 1  % of Total / Decay Time (s) | POPULATION 2  % of Total / Decay Time (s) |
| --- | --- | --- |
| - oxygen scavenging buffer | 31 % / 5 s | 69 % / 17 s |
| + oxygen scavenging buffer | 10 % / 195 s | 90 % / 1100 s |

## Localization of individual enzymes in a single-molecule (SM) fluorescence image sequence using DAOSTORM

A typical single-molecule fluorescence image of Cy5-labeled enzymes can contain thousands of individual enzymes seen as spots within a field of view (Figure S.3a). Localization of the spots in the frames and measuring the lifetime of each individual spot throughout the time of collection is a challenge. In previous imaging studies of binding lifetimes of individual cellulases on cellulose, the images were analyzed manually, where the lifetimes of handpicked spots were collected from the image stacks (e.g. Figure S.3 d-f). Manual processing of the large data sets is time-intensive and subject to user-bias in the selection of ‘representative’ spots. Moreover, the specificity of intact Cel7A binding to cellulose surfaces resulted in crowding on some regions such that existing plugins in ImageJ (e.g. ThunderSTORM (3)) proved insufficient at fully resolving individual enzymes. To overcome these limitations, an automated data analysis process was developed in this study. DAOSTORM, an algorithm based on localizing x and y coordinates of individual stars in crowded stellar fields, applies the point-spread function (PSF) of the brightest isolated spots to identify and localize spots in a given field of view. Localized spots are deleted from the data set and the localization process is iterated with the remaining spots until no more new spots are identified (4). Figures S.3b and c demonstrate the application of DAOSTORM in the current experiments to localize individual enzymes in a crowded field of view. Localized spots in all the frames of each image stack were compiled to generate datasets of binding time distributions of all the enzymes over the course of the experiments.


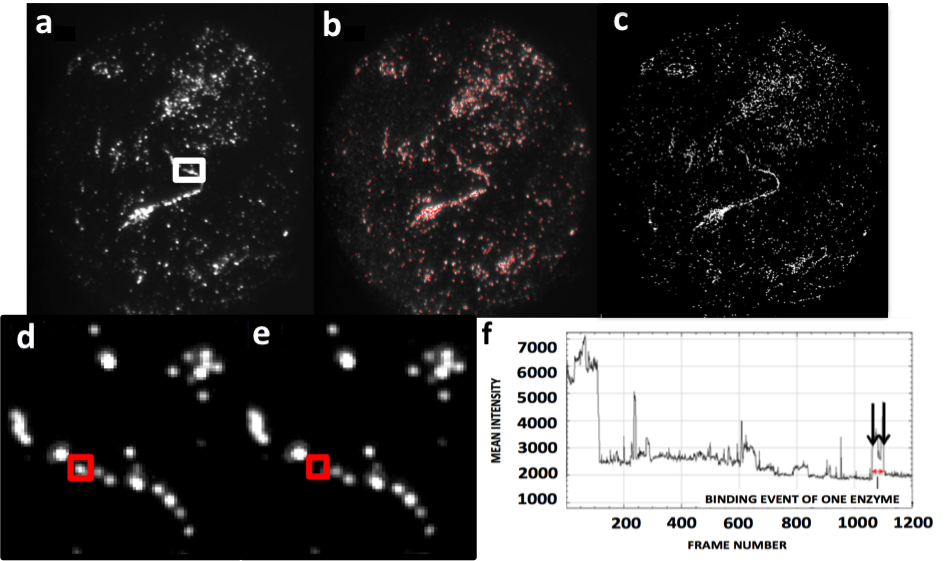


Figure S.3 (a) Raw image of 54×54 μm^2^ field of view containing Cy5-labeled Cel7A bound to algal cellulose. (b) Image showing the centroids (red crosses) of the individual enzyme molecules as detected by DAOSTORM software overlaid on (a). (c) Super resolution image reconstructed from the DAOSTORM output. (d)-(f) Zoomed in portion of box shown in (a), demonstrate manual determination of the binding lifetime of individual enzymes as adopted by earlier studies. (d) One enzyme molecule (in 5×5 pixel area outlined by a red square) appeared (bound) in frame 1055 and (e) disappeared (unbound) 101 frames later in frame 1156. (f) Intensities associated with the appearance and disappearance of the enzyme in (d) and (e) (arrows) are observed in the mean intensity profile of the 5×5 pixel area. This manual process is repeated many times to obtain binding time distributions of the enzymes within a given experiment.

The raw data, collected as a sequence of 16-bit unsigned integer images (Winview SPE format), were converted to 32-bit signed floating point using ImageJ (Version, 1.49, NIH). The 32-bit image stack was divided by the multiplicative gain (typically 15-30) of the EMCCD camera and offset by 10000 to facilitate the DAOSTORM analysis. This image stack was stored as a sequence of FITS (Flexible Image Transport System) formatted images. The image sequence was then combined into a single FITS file as required for input into the DAOSTORM software. DAOSTORM, written as a Python module, contains tools to calculate the point spread function (PSF) from the brightest spots that are well isolated. The frame number for obtaining the PSF file, the standard deviation of the background noise and minimum and maximum data values are input parameters for the algorithm to generate the PSF. The PSF is applied to localize every point in every frame in a given movie, producing an output file that contains the position (x,y) coordinates and frame numbers of each of the spots found by DAOSTORM.

## Obtaining Binding lifetimes of individual enzyme molecules using output from DAOSTORM

The output from the DAOSTORM analysis was corrected to account for lateral drift of the microscope stage using Image J software (Version 1.49, NIH) using the method of cross correlation implemented in the ThunderStorm ImageJ plugin (3). A custom algorithm, implemented in Python, was used to compile and generate histograms of the cellulase binding times on cellulose (5). A brief description of this algorithm is given here. A mask around the fibril was constructed using the super-resolution image to ensure only spots binding to cellulose were chosen for analysis (e.g. Figure 3 in the main text). At the start of the iterative process, all points localized in the first frame are collected into a distinct array. In subsequent iterations through the image stack, the x and y coordinates of points in each frame are compared to those from the previous frame in the array by calculating the inter-frame distance between the two points. If the inter-frame distance is greater than a specified number of pixels (2 pixels was employed), the point is assigned as a new point in the distinct array and the first frame number is set. If the inter-frame distance is less than two pixels, the point location is deemed unchanged and the ending frame number is incremented by 1. Once all the points in all the frames are processed, the data set is binned into 10-second interavals to generate the binding time histogram. Histograms generated for the current study are shown in Figure S 4.

## Multi-exponential fitting of the binding time histograms

Several fitting procedures were tested to best fit the exponential decay data that were obtained from the single molecule experiments. The binding time histograms were fit to multicomponent exponential decay equations with up to four exponents using GenCurvefit (6) that uses differential evolution (Genetic optimization) and fits by minimizing a user defined cost function in Igor Pro (Version 6.34A, Wavemetrics) (7):

$f\left( t \right)=A_{1}\exp\frac{-t}{\boldsymbol{\tau}_{1}}$ +$A_{2}\exp\frac{-t}{\boldsymbol{\tau}_{2}}+A_{3}\exp\frac{-t}{\boldsymbol{\tau}_{3}}$ $A_{4}\exp\frac{-t}{\boldsymbol{\tau}_{4}}$. (Eq. S. 1)

Where, A_1_, A_2_, A_3_ and A_4_ represent the amplitude each fraction with characteristic binding times of τ_1_, τ_2,_ τ_3,_ and τ_4_ (s), respectively.

The Poisson deviance cost function was input as the user defined function (8):

$P_{d}=\frac{2}{N}\sum_{i=1}^{N} [D_{i}\ln(\frac{D_{i}}{F_{i}})+F_{i}-D_{i}]$ (Eq. S. 2)

Where D_i_ is the observed value, F_i_ is the fit value and N is the number of points. The reduced Poisson Deviance was calculated by:

$P_{dr}=\frac{P_{d}\times N}{(N-N_{p}-1)}$ (Eq. S. 3)

Where N_p_ is the number of fit parameters.

The reduced Poisson deviance (close to 1) and residual plots for each of the fits were used as metrics to carefully select the optimal number of exponents to describe the data (Table S. 1).

Table S. 1: The Poisson deviance (Eq. S. 2) and reduced Poisson deviance (Eq. S.2) values obtained from multiexponential fitting (Eq. S.1) of the binding time histograms (N = 251). Generally, the fit with the least number of exponents with P_dr_ ~ 1 was taken as the best fit to the data.

| Enzyme | Raw data filename^a^ | No. of Exponents | P_d_ | P_dr_ | Best fit |
| --- | --- | --- | --- | --- | --- |
| **WT_intact_** | 111130 | 1 | 3016 | 12.1 | 3-exp |
|  |  | 2 | 905 | 3.6 |  |
|  |  | **3** | **229** | **0.9** |  |
|  |  | 4 | 232 | 1.0 |  |
| **WT_core_** | 123032 | 1 | 33001 | 132.0 | 3-exp |
|  |  | 2 | 2037 | 8.2 |  |
|  |  | **3** | **190** | **0.8** |  |
|  |  | 4 | 261 | 1.1 |  |
|  | 143742 | 1 | 5574 | 22.3 |  |
|  |  | 2 | 2246 | 9.1 |  |
|  |  | **3** | **233** | **0.9** |  |
|  |  | 4 | 255 | 1.0 |  |
|  | 133451 | 1 | 30131 | 120.5 |  |
|  |  | 2 | 30131 | 121.5 |  |
|  |  | **3** | **216** | **0.9** |  |
|  |  | 4 | 263 | 1.1 |  |
| **E212Q_intact_** | 170336 | 1 | 4636 | 18.5 | 2-exp |
|  |  | **2** | **117** | **0.5** |  |
|  |  | 3 | 114 | 0.5 |  |
|  |  | 4 | 114 | 0.5 |  |
|  | 160828 | 1 | 3163 | 12.7 |  |
|  |  | **2** | **186** | **0.8** |  |
|  |  | 3 | 183 | 0.7 |  |
|  |  | 4 | 183 | 0.8 |  |
| **E212Q_core_** | 190922 | 1 | 16009 | 64.0 | 3-exp |
|  |  | 2 | 16009 | 64.6 |  |
|  |  | **3** | **231** | **0.9** |  |
|  |  | 4 | 221 | 0.9 |  |
|  | 181309 | 1 | 12941 | 51.8 |  |
|  |  | 2 | 213 | 0.9 |  |
|  |  | **3** | **166** | **0.7** |  |
|  |  | 4 | 180 | 0.7 |  |

^a^ Data files containing the raw data (movies) corresponding to this analysis are published in (9) and identifiable by the filename provided.

Table S. 2: Fitting parameters from the best-fit model to a multiexponential decay equation (Eq S.1). Each data set with N = 251 (histograms of binding times in 10 s bins with 251 bins) was fit to one, two, three and four exponential decay equations. The number of populations that best represent the data were decided from fits that resulted in P_dr_ closest to 1 are shown in this table.

| **Enzyme** | **Substrate** | **Raw data filename^a^** | **Population 1** | | | | **Population 2** | | | | **Population 3** | | | | **P_dr_^c^** | **# Enzymes Counted** |
| --- | --- | --- | --- | --- | --- | --- | --- | --- | --- | --- | --- | --- | --- | --- | --- | --- |
|  |  |  | **A_1_** | **error^b^** | **τ_1_ (s)** | **error^b^** | **A_2_** | **error^b^** | **τ_2_ (s)** | **error^b^** | **A_3_** | **error^b^** | **τ_3_ (s)** | **error^b^** |  |  |
| **WT_intact_** | Fibril | 101907^d^ | 7197 | 144 | **15** | 0.01 | 294 | 13.6 | **155** | 0.01 | - | - | **-** | - | 1.4 | 12283 |
|  | Fibril | 111130 | 1729 | 86 | **15** | 0.02 | 29 | 4 | **187** | 0.05 | 0.6 | 0.3 | **1260** | 0.15 | 0.9 | 2452 |
|  | Background |  | 1051 | 23 | **9** | 0.008 | 25 | 0.0 | **26** | 0.03 | - | - | - | - | 0.03 | 528 |
| **WT_core_** | Fibril | 123032 | 4230 | 148 | **13** | 0.01 | 161 | 18 | **85** | 0.04 | 14 | 3 | **376** | 0.034 | 0.8 | 5290 |
|  | Background |  | 1044 | 0.0 | **9** | 0.009 | 16 | 2 | **59** | 0.02 | - | - | - | - | 0.1 | 585 |
|  | Fibril | 143742 | 4470 | 144 | **15** | 0.01 | 171 | 19 | **104** | 0.04 | 9 | 2.8 | **438** | 0.05 | 0.9 | 6615 |
|  | Background |  | 599 | 24 | **10** | 0.01 | 32 | 4 | **45** | 0.02 | - | - | - | - | 0.1 | 469 |
|  | Fibril | 133451 | 3590 | 127 | **14** | 0.01 | 82 | 10 | **108** | 0.05 | 15 | 3.9 | **354** | 0.04 | 0.9 | 4819 |
| **E212Q_intact_** | Fibril | 170336 | 619 | 35 | **13** | 0.02 | 2 | 0.4 | **332** | 0.04 | - | - | - | - | 0.5 | 642 |
|  | Background |  | 768 | 18.5 | **9** | 0.008 | 17 | 0.3 | **38** | 0.024 | - | - | - | - | 0.04 | 464 |
|  | Fibril | 160828 | 308 | 0.3 | **17** | 0.03 | 4 | 0.6 | **368** | 0.04 | - | - | - | - | 0.8 | 532 |
|  | Background |  | 915 | 0.4 | **10** | 0.01 | 5 | 1.2 | **70** | 0.05 | - | - | - | - | 0.1 | 580 |
| **E212Q_core_** | Fibril | 190922 | 1699 | 87 | **15** | 0.02 | 57 | 12 | **114** | 0.06 | 1.1 | 0.3 | **870** | 0.06 | 0.9 | 2551 |
|  | Background |  | 388 | 0.2 | **11** | 0.01 | 6 | 1.2 | **66** | 0.04 | - | - | - | - | 0.1 | 291 |
|  | Fibril | 181309 | 1375 | 65 | **15** | 0.02 | 54 | 14 | **94** | 0.09 | 5.5 | 1.89 | **421** | 0.06 | 0.7 | 2138 |
|  | Background |  | 958 | 28 | **9** | 0.01 | 16 | 2 | **49** | 0.0 | - | - | - | - | 0.1 | 499 |

^a^ Data files containing the raw data (movies) corresponding to this analysis are published in (9) and identifiable by the filename provided.

^b^ Estimated standard deviation of the fit coefficients.

^c^ Reduced Poisson Deviance parameter (Eq S. 3).

^d^Results from this analysis was not considered in this study because the binding data were collected using a higher laser power of 2mW rather than 0.2 mW that was used for all the other data sets.

##
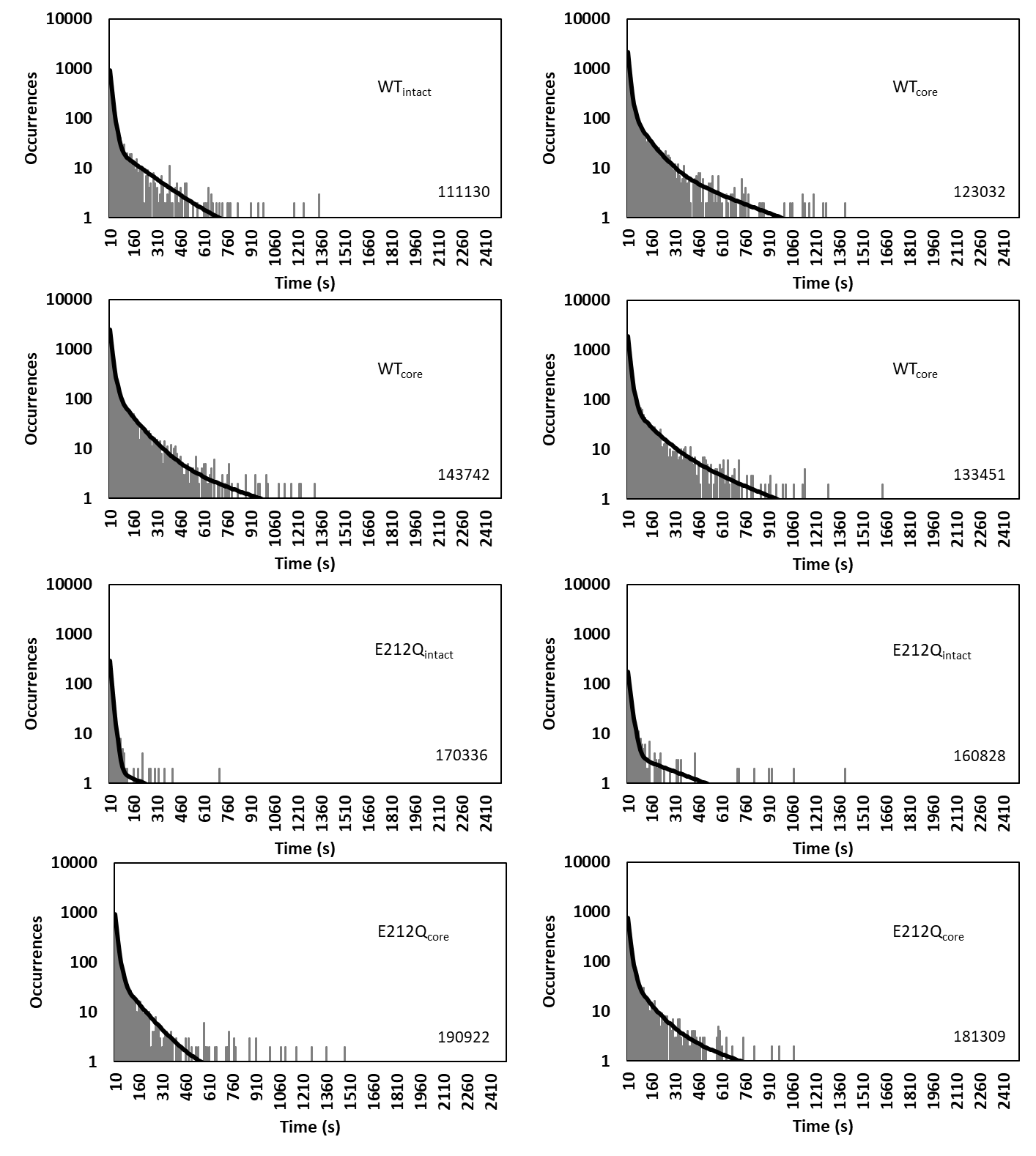


Figure S 4. Multiexponential fits to histograms of enzyme binding to cellulose fibrils. Gray bars show counts of enzymes in the given residence time bin and black lines are multiexponential decay equations using parameters in Table S. 2. Raw data filenames are provided in the bottom right of each graph. The histograms were generated from data collected over 2500 s.

## Binding of Cy5-labeled Cel7A and Cel7A CD on phosphoric acid swollen cellulose (PASC)

WT_intact­_ and WT_core_ binding to PASC is shown in Figure S 4.


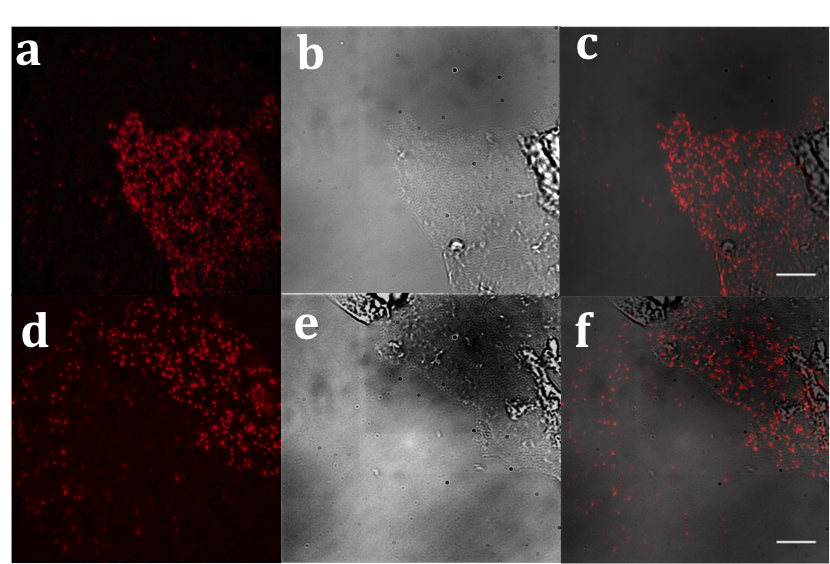


Figure S 5. (a-c) Binding of WT_intact_ and (d-f) WT_core_ on PASC. (a),(d) summed fluorescence data showing the binding of single fluorescent enzyme molecules. (b),(e) white light images of the same regions as (a), (d). (c) and (f) overlay of the fluorescence data on the white light image. Scale bar is 8 μm.

An earlier study by Nakamura et al. showed that the specific activity of the W40 mutant of Cel7A and its catalytic core (W40_core_) was 2.2 times higher on PASC than on crystalline algal cellulose. The W40 mutation at the entrance of the active site disables the enzyme’s ability to uptake a chain into its active site, thus inhibiting complexation. While this mutation drastically lowered the digestibility of crystalline cellulose, they found that it did not affect PASC hydrolysis rates (10). The authors suggested that the free chain ends at the surface of cellulose are longer for PASC, making it easy to complex and hence compensating for the mutation. Our studies suggest that the core enzymes complex PASC as easily as the intact enzymes despite the lack of CBM and linker.

## Automating the analysis of single-molecule (SM) image stacks for unbiased determination of binding lifetimes of cellulases on cellulose

Previously, Jung et al. (11) reported two populations of Cel7A binding to cellulose with binding lifetimes of 30 s (84 %) and 173 s (16 %) by the same SM imaging method used in this study. The residence times were similar to specific binding times observed for the WT_intact_ and E212Q_intact_, but Jung et al. did not report a non-specific binding fraction with short binding lifetimes of 8 – 10 s observed in these experiments. In a separate SM imaging study, Shibafuji et al. (12) reported a biexponential decay fit to binding histograms of Cel7A on AC with average binding lifetime of 1-4 s (52 %) and 8 s (44 %). It was interesting to note that this group did not report a fraction with > 100 s binding times. A side-by-side comparison of the results from the three SM imaging experiments using the same enzyme with similar cellulosic substrates emphasizes the disparity that can arise from the processing of SM imaging data. Jung et al. (11) and Shibafuji et al. (12) compiled binding histograms from SM image stacks by hand, arbitrarily selecting several hundred spots for which binding times were manually recorded. The automated spot finding algorithm utilized in this experiment facilitated unbiased determination of binding times for all identifiable spots that appeared and disappeared throughout the window of observation. Binding times from our datasets overlap the reported binding times of both Jung et al. and Shibafuji et al. with some explainable differences. We did not observe the 1 s residence time fraction in our studies because of the added precautions to control for photostability issues using the oxygen-scavenging buffer and trolox to control for bleaching and blinking of the fluorophores (2). Similar precautions were noted in Jung et al., but no control for blinking of the fluorophores was reported by Shibafuji et al. As best as we can determine, Jung et al. may have only selected spots that resided longer than 10 s, thus only reporting on the specifically bound cellulase populations. Thus we conclude that the spot-finding algorithm based method used in the current study provides the more complete and objective analysis of the binding times of cellulases on cellulose.

Table 2 Binding lifetimes (τ) of Cy5-labeled Cel7A on AC measured by SM imaging in literature.

|  | Relative Fraction | | τ (s)  (k_off,_ s^-1^)^1^ | Relative Fraction | τ (s)  (k_off,_ s^-1^)^1^ | Relative Fraction | τ (s)  (k_off,_ s^-1^)^1^ | Relative Fraction | τ (s)  (k_off,_ s^-1^)^1^ |
| --- | --- | --- | --- | --- | --- | --- | --- | --- | --- |
| Shibafuji et al. 2014 | | 52 % | 1  (0.9) | 48 % | 8  (0.2) |  |  | |  |
|  |  | Non-productive^2^ | | Productive^2^ | |  |  |  |  |
| Jung et al. 2013 | |  |  |  |  | 84% | 30  (0.03) | 16% | 173  (0.005) |
|  |  |  |  |  |  | Non-productive^2^ | | Productive^2^ | |
| Current work | |  |  | 81 % | | 15  (0.067) | | 19 % | > 200 s |

^1^Specific dissociation rates (k_off_) in s^-1^ were determined from the inverse of the residence times (τ) in s.

^2^Productive and non-productive binding assignments made by the authors of the study.

Shibafuji et al. attributed the 1 s residence time to binding of Cel7A to cellulose by its CBM and the longer, 8 s residence time to binding by its catalytic domain (12). The challenge is that these experiments neither directly probed the binding of the isolated catalytic domain fractions on cellulose nor measured catalysis events directly by the bound cellulases.

## References

1. Stahlberg J, Divne C, Koivula A, Piens K, Claeyssens M, Teeri TT, et al. Activity Studies and Crystal Structures of Catalytically Deficient Mutants of Cellobiohydrolase I from *Trichoderma reesei*. J Mol Biol. 1996;264:337–49.

2. Aitken CE, Marshall RA, Puglisi JD. An Oxygen Scavenging System for Improvement of Dye Stability in Single-Molecule Fluorescence Experiments. Biophysical Journal. 2008;94(5):1826–35.

3. Ovesný M, Křížek P, Borkovec J, Svindrych Z, Hagen GM. ThunderSTORM: a comprehensive ImageJ plug-in for PALM and STORM data analysis and super-resolution imaging. Bioinformatics. 2014 Aug 15;30(16):2389–90.

4. Holden SJ, Uphoff S, Kapanidis AN. DAOSTORM: an algorithm for high- density super-resolution microscopy. Nat Meth. 2011 print;8(4):279–80.

5. Mudinoor AR. Surface Interactions of *Trichoderma reesei* Cel7A and Its Variants on Cellulose. University of California, Davis; 2018.

6. Nelson A. GenCurvefit (Igor Exchange). Australian Nuclear Science and Technology Organization; 2007.

7. Wormington M, Panaccione C, Matney KM, Bowen DK. Characterization of structures from X-ray scattering data using genetic algorithms. Bowen DK, Tanner BK, editors. Philosophical Transactions of the Royal Society of London Series A: Mathematical, Physical and Engineering Sciences. 1999 Oct;357(1761):2827–48.

8. Steinbach PJ. Filtering artifacts from lifetime distributions when maximizing entropy using a bootstrapped model. Analytical Biochemistry. 2012 Aug;427(1):102–5.

9. Jeoh T, Goodwin PM, Mudinoor AR. Single-Molecule Image Sequences of Trichoderma reesei Cel7A and Variants on Cellulose Fibrils [Internet]. UC Davis; 2020 Feb. Available from: https://doi.org/10.25338/B8ZC80

10. Nakamura A, Tsukada T, Auer S, Furuta T, Wada M, Koivula A, et al. The Tryptophan Residue at the Active Site Tunnel Entrance of Trichoderma reesei Cellobiohydrolase Cel7A Is Important for Initiation of Degradation of Crystalline Cellulose. J Biol Chem. 2013 May 10;288(19):13503–10.

11. Jung J, Sethi A, Gaiotto T, Han JJ, Jeoh T, Gnanakaran S, et al. Binding and movement of individual Cel7A cellobiohydrolases on crystalline cellulose surfaces revealed by single-molecule fluorescence imaging. J Biol Chem. 2013 Jul 1;288(33):24164–72.

12. Shibafuji Y, Nakamura A, Uchihashi T, Sugimoto N, Fukuda S, Watanabe H, et al. Single-molecule Imaging Analysis of Elementary Reaction Steps of Trichoderma reesei Cellobiohydrolase I (Cel7A) Hydrolyzing Crystalline Cellulose Iα and IIII. J Biol Chem. 2014 May 16;289(20):14056–65.
